# Cost-effective methylome sequencing of cell-free DNA for accurately detecting and locating cancer

**DOI:** 10.1038/s41467-022-32995-6

**Published:** 2022-09-29

**Authors:** Mary L. Stackpole, Weihua Zeng, Shuo Li, Chun-Chi Liu, Yonggang Zhou, Shanshan He, Angela Yeh, Ziye Wang, Fengzhu Sun, Qingjiao Li, Zuyang Yuan, Asli Yildirim, Pin-Jung Chen, Paul Winograd, Benjamin Tran, Yi-Te Lee, Paul Shize Li, Zorawar Noor, Megumi Yokomizo, Preeti Ahuja, Yazhen Zhu, Hsian-Rong Tseng, James S. Tomlinson, Edward Garon, Samuel French, Clara E. Magyar, Sarah Dry, Clara Lajonchere, Daniel Geschwind, Gina Choi, Sammy Saab, Frank Alber, Wing Hung Wong, Steven M. Dubinett, Denise R. Aberle, Vatche Agopian, Steven-Huy B. Han, Xiaohui Ni, Wenyuan Li, Xianghong Jasmine Zhou

**Affiliations:** 1 Department of Pathology and Laboratory Medicine, David Geffen School of Medicine, University of California at Los Angeles, Los Angeles, CA 90095 USA; 2 EarlyDiagnostics, Inc., 570 Westwood Plaza, Los Angeles, CA 90095 USA; 3 Department of Quantitative and Computational Biology, University of Southern California, Los Angeles, CA 90089 USA; 4 The Eighth Affiliated Hospital, Sun Yat-Sen University, Shenzhen, China; 5 Department of Microbiology, Immunology and Molecular Genetics, University of California at Los Angeles, Los Angeles, CA 90095 USA; 6 Department of Surgery, University of California at Los Angeles, Los Angeles, CA 90095 USA; 7 Department of Molecular and Medical Pharmacology, David Geffen School of Medicine, University of California at Los Angeles, Los Angeles, CA 90095 USA; 8 Westlake High School, 100N Lakeview Cyn Road, Westlake Village, CA 91362 USA; 9 Department of Medicine, David Geffen School of Medicine, University of California at Los Angeles, Los Angeles, CA 90095 USA; 10 Department of Radiological Sciences, David Geffen School of Medicine, University of California at Los Angeles, Los Angeles, CA 90095 USA; 11 Jonsson Comprehensive Cancer Center, University of California at Los Angeles, Los Angeles, CA 90095 USA; 12 VA Greater Los Angeles Health Care System, Los Angeles, CA 90073 USA; 13 Institute for Precision Health, University of California at Los Angeles, Los Angeles, CA 90095 USA; 14 Institute for Quantitative and Computational Biosciences, University of California at Los Angeles, Los Angeles, CA 90095 USA; 15 Department of Statistics, Stanford University, Stanford, CA 94305 USA; 16 Department of Biomedical Data Science, Stanford University, Stanford, CA 94305 USA

**Keywords:** Cancer screening, Software, Computational biology and bioinformatics, Statistical methods, Diagnostic markers

## Abstract

Early cancer detection by cell-free DNA faces multiple challenges: low fraction of tumor cell-free DNA, molecular heterogeneity of cancer, and sample sizes that are not sufficient to reflect diverse patient populations. Here, we develop a cancer detection approach to address these challenges. It consists of an assay, cfMethyl-Seq, for cost-effective sequencing of the cell-free DNA methylome (with > 12-fold enrichment over whole genome bisulfite sequencing in CpG islands), and a computational method to extract methylation information and diagnose patients. Applying our approach to 408 colon, liver, lung, and stomach cancer patients and controls, at 97.9% specificity we achieve 80.7% and 74.5% sensitivity in detecting all-stage and early-stage cancer, and 89.1% and 85.0% accuracy for locating tissue-of-origin of all-stage and early-stage cancer, respectively. Our approach cost-effectively retains methylome profiles of cancer abnormalities, allowing us to learn new features and expand to other cancer types as training cohorts grow.

## Abstract (web summary)

Early cancer detection by cell-free DNA (cfDNA) is challenged by the low amount of tumour DNA in cfDNA, tumour heterogeneity and the small patient cohorts. Here, the authors develop a method, cfMethyl-Seq, for cost-effective methylome profiling of cfDNA and for detecting and locating cancer.

## Introduction

Detecting cancer before metastasis is the key to fighting it successfully. Recently, cell-free DNA (cfDNA) has drawn attention for its utility in early cancer detection^1–3^. Specifically, cfDNA methylation has been shown to be a highly promising feature, capable of not just detecting cancer but also locating its Tissue of Origin (TOO)^2,4–10^. Despite its great promise, cfDNA-based cancer detection faces some major challenges: (1) the fraction of tumor cfDNA in the blood of early-stage cancer patients can be very low, (2) the signatures of cfDNA aberrations from diverse cancer types, subtypes, stages and etiologies are heterogeneous; and (3) the currently available sample sizes are small compared to the diversity of diseases and the patient population (age, gender, ethnicity, and comorbidity), especially for pan-cancer detection.

To address the challenge of a very low tumor fraction in cfDNA, a methylome test can exploit as many cfDNA fragments in the blood as possible, while traditional small-panel, deep-sequencing approaches only capture a small proportion of all tumor cfDNA fragments, likely resulting in false negatives. To address the molecular heterogeneity of cancer, a methylome-based test can cover the broad landscape of methylation markers of different cancer types and etiologies. To address the challenge of limited sample sizes currently available, a methylome-based test can learn and exploit newly significant markers as the training cohort grows, while for small panel-based approaches the same set of markers are measured in all new patient samples, and no new markers can be discovered and validated. Despite the advantages offered by cfDNA methylome analysis, the commonly used whole-genome bisulfite sequencing (WGBS) is too expensive to be used in clinical applications.

Here, we present an integrated experimental and computational system for the accurate and affordable detection of cancer. It consists of (1) a cost-effective experimental assay, named cell-free DNA Methylome Sequencing (cfMethyl-Seq), for genome-wide methylation profiling of cfDNA, and (2) a computational method to extract four types of cfDNA methylation features (cancer-specific and tissue-specific hypermethylation and hypomethylation markers), and perform ensemble learning for detecting and locating cancer. Our experimental approach cfMethyl-Seq performs methylation profiling of CpG-rich regions, which occupy only ~3% of the whole genome. Therefore, cfMethyl-Seq is dramatically cheaper than WGBS, while still capturing most (>90%) CpG islands. To fully characterize the cfDNA methylome from cfMethyl-Seq data, we identify four types of methylation features, cancer(tissue)-specific hyper(hypo)methylation features, which are then integrated via an ensemble learning model. We apply cfMethyl-Seq to cfDNA samples of 408 individuals, including 217 patients with colon, liver, lung or stomach cancer, and 191 individuals without cancer (including patients with various other diseases). 54% of the cancer patients are in early stages (I and II), and in particular 73% of liver cancer patients are in Stage I. We demonstrate the performance on two tasks: (1) Detecting cancer: we achieve an overall AUC of 0.974 (95% CI: 0.926 to 0.998), with the overall sensitivity of 80.7% (95% CI: 68.6% to 90.7%) at the specificity of 97.9%. The sensitivities of the individual cancer types range from 75.9% to 92.3%. (2) Locating cancer: the prediction of the tumor’s TOO yields an accuracy of 89.1% (95% CI: 73.9% to 96.9%). We perform extensive validations, e.g. cross-batch validation, cross-source validation, age-matched validation, and independent validation, to confirm the robustness of our results. In this work, we develop a cost-effective cfDNA methylome sequencing method, cfMethyl-Seq, and demonstrate its power in detecting and locating cancer. Our results show that among individual methylation features, cancer-specific hypermethylation exhibits the highest power in detecting cancer while cancer-specific hypomethylation is most informative for TOO prediction. Encouragingly, we demonstrate that with increasing sample sizes, the detection power of our system continues to improve.

## Results

### Cost-effective cell-free DNA methylome sequencing (cfMethyl-Seq)

We developed the cfMethyl-Seq technique to address the challenge of cost-effectively profiling genome-wide methylation in cfDNA. Note that the traditional method, reduced representation bisulfite sequencing (RRBS), can also enrich CpG-rich regions—but only from intact genomic DNA, not from cfDNA. RRBS employs restriction enzymes to cut intact DNA into small fragments in regions with high CpG content, then size-selects these fragments to enrich for CpG-dense regions. Because cfDNA is already fragmented, the size selection step of RRBS will select nearly all cfDNA and fail to enrich the CpG-dense regions.

**Fig. 1:**
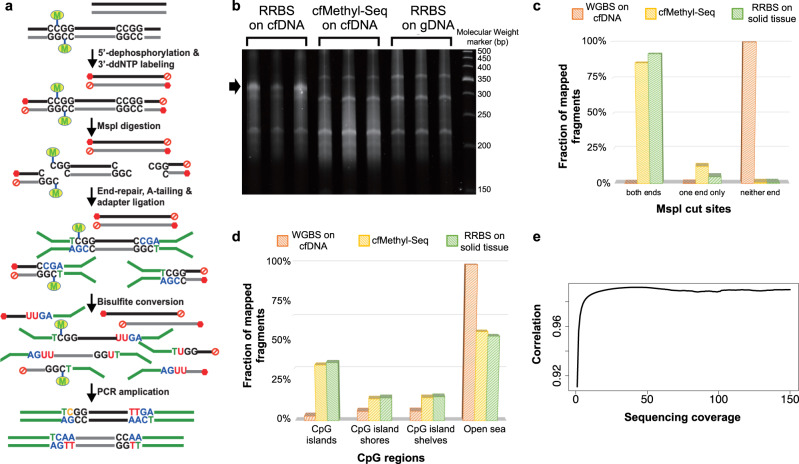
cfMethyl-Seq assay. **a** Diagram of the cfMethyl-Seq protocol. **b** Typical TBE-UREA PAGE image of cfMethyl-Seq libraries made from cfDNA, compared with conventional RRBS with cfDNA or intact genomic DNA as input material. The non-specific ligation product from cfDNA fragments with the conventional RRBS protocol is indicated by an arrow. This technical validation experiment was repeated independently twice and showed similar results. For cfMethyl-Seq assays generating data for analysis, each sample was constructed into library without replicate. **c** The percentage of reads with MspI sites on both ends, on only one end, and on neither end from WGBS assay, our cfMethyl-Seq assay, and RRBS assay on cfDNA. Source data are provided as a Source Data file. **d** The percentage of mapped fragments that fall in CpG islands, CpG island shores, CpG island shelves, and open sea regions is shown for cfMethyl-Seq libraries, RRBS libraries, and WGBS libraries on cfDNA. Source data are provided as a Source Data file. **e** Methylation concordance between a genomic DNA sample sequenced with RRBS, and sheared and sequenced with cfMethyl-Seq, increases with depth of coverage. Pearson correlation (y-axis) of the methylation rate (beta value) in the two datasets was calculated on the CpG sites that are covered by both datasets at minimum depth of coverage specified on the x-axis. Source data are provided as a Source Data file. Abbreviations: RRBS Reduced representation bisulfite sequencing.

In the cfMethyl-Seq process, as shown in Fig. 1a, we first block both ends of all cfDNA fragments by dephosphorylating their 5’-ends and adding ddNTP to their 3’-ends. After digestion with the restriction enzyme MspI (cut site C | CGG), only those cfDNA fragments with two or more CCGG sites will be able to ligate to adapters. Sequencing these fragments results in a library that is highly enriched in CpG sites. Note that our adapters contain duplex unique molecular identifiers (UMIs). This is necessary because enzymatic digestion causes many fragments to map to the same start and end locations, creating challenges for conventional PCR deduplication^11^.

The cfMethyl-Seq libraries built on cfDNA show characteristic bands, with insert lengths of 68 bp, 135 bp, and 203 bp (Fig. 1b and Supplementary Fig. S1). This structure is a result of MspI digestion of Alu repeat elements. These bands are positioned similarly to those seen in conventional RRBS libraries prepared from solid tissue (Fig. 1b). However, the strongest band in conventional RRBS libraries prepared from cfDNA (Fig. 1b) is located around 167 bp: the characteristic size of full-length cfDNA fragments without MspI digestion. This confirms that size selection in RRBS on cfDNA fails to achieve the desired enrichment of CpG-dense fragments.

We compared our cfMethyl-Seq libraries to cfDNA WGBS libraries, as well as to the conventional RRBS protocol performed on solid tissues. On average, 85.7% of reads in 408 cfMethyl-Seq libraries have MspI cutting sites on both ends, compared to 91.8% of reads in the 251 RRBS libraries and only 0.00006% of reads in 37 WGBS libraries of cfDNA (Fig. 1c). Consequently, 34.11%, 12.38%, and 13.14% of cfMethyl-Seq reads come from CpG island, shore, and shelf regions, compared to 33.65%, 13.35%, and 14.04% of conventional RRBS reads, respectively (Fig. 1d). As for the WGBS cfDNA libraries, only 2.66% of reads come from CpG islands but most (88.32%) come from uninformative “open sea” regions (Fig. 1d). That is, cfMethyl-Seq offers a 12.8× enrichment over WGBS in CpG islands. Comparisons between traditional RRBS libraries on solid tissue and cfMethyl-Seq libraries on the same tissue’s sheared gDNA show that cfMethyl-Seq can obtain similar methylation levels, with correlation significantly increasing as coverage increases up to 10x (Fig. 1e). For CpG sites with more than 10x coverage, the Pearson correlation between methylation levels in cfMethyl-Seq on sheared gDNA and RRBS on intact gDNA is 0.987 (*P* value <2.2e-16) for the same sample. In replicate samples, the Pearson correlation of depth of coverage is >0.98 (*P* value <2.2e-16) and the Pearson correlation of methylation levels for CpG sites with more than 10x coverage is >0.99 (*P* value <2.2e-16).

### Discovery of methylation markers for cancer detection and tissue-of-origin prediction

**Fig. 2:**
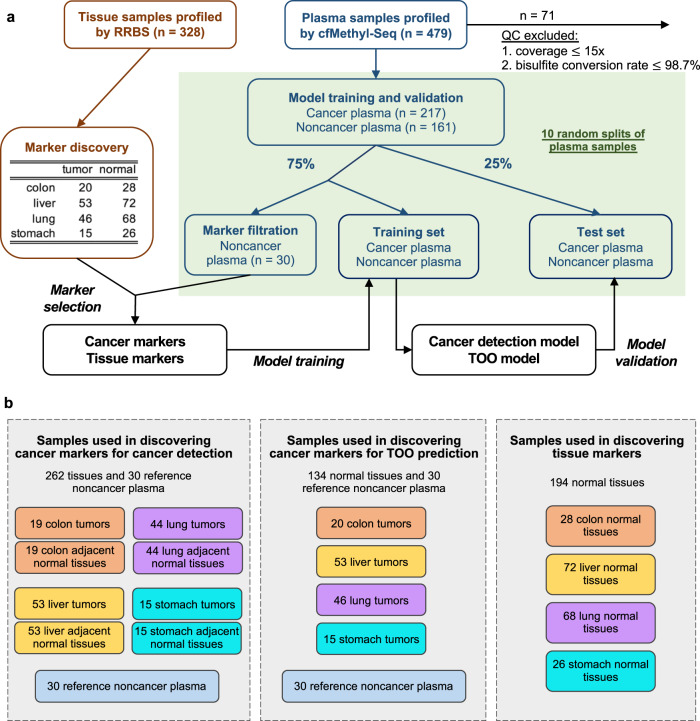
Study design and overview of the computational method. **a** Overview of the sample usage for marker discovery, model training, and validation. All tissue samples are used for marker discovery, and all plasma samples are randomly split into three sets, used for marker discovery, training, and validating the predictive model. The plasma sample split is repeated 10 times and the prediction performance is averaged over the 10 runs. **b** Details of sample usage for marker discovery. Different types of methylation markers were discovered by using different samples. Note that 30 reference noncancer plasma samples (in blue boxes) correspond to “marker filtration” in **a**. Abbreviations: TOO tissue of origin.

We generated RRBS data for 328 solid tissues for marker discovery, including 131 pairs of solid tumors and their adjacent normal tissues, for colon (19 pairs), liver (53 pairs), lung (44 pairs), and stomach (15 pairs) cancer (see Fig. 2a for the overall study design, and Fig. 2b for detailed sample usage for marker discovery). The unit regions of our methylation markers are defined to be genomic locations that are between two adjacent MspI cutting sites (i.e., between two CCGG sites) and that are less than 350 bp (the average region size is 117 bp). Using our read-based marker discovery method (see Methods and Supplementary Information S1–S3), we identified cancer-specific markers that significantly differ between solid tumors and their adjacent normal tissues. In addition, we required the cancer-specific markers to have differential methylation between solid tumors and the cfDNA of 30 reference noncancer individuals. The 30 reference noncancer individuals were randomly drawn in each validation run, resulting in slightly different cancer-specific markers across runs. In each run, we merged the identified markers from all cancer types, and across 10 runs we obtained an average of 23,748 cancer-specific hypermethylation markers (Supplementary Table S1) and 28,197 cancer-specific hypomethylation markers for cancer detection (Supplementary Table S2). Moreover, for TOO prediction we identified the markers that significantly differ between any two solid tumor types as well as between solid tumors and the 30 reference noncancer plasma samples, by using RRBS data of 134 solid tumors for colon (20), liver (53), lung (46), and stomach (15) cancer. Similarly, in each run, we merged the identified markers from all pairwise comparisons, and across 10 runs we obtained on average 30,474 cancer-specific hypermethylation (Supplementary Table S3) and 33,890 cancer-specific hypomethylation markers (Supplementary Table S4) for cancer TOO prediction. These markers are referred to as cancer-specific methylation markers for cancer TOO prediction.

Since organs that contain tumors undergo increased cell death and therefore yield an elevated quantity of cfDNA^12–14^, tissue-specific cfDNA deconvolution can aid in both detecting cancer and predicting its TOO. Using RRBS data of 194 solid normal tissues (28 colon tissues, 72 liver tissues, 68 lung tissues, and 26 stomach tissues (Fig. 2b)), we identified markers that significantly differ between any two types of solid normal tissues, and merged the identified markers from every two tissue types, resulting in 7547 tissue-specific hypermethylation (Supplementary Table S5) and 7212 tissue-specific hypomethylation markers (Supplementary Table S6) that can differentiate between pairs of tissue types.

To demonstrate the differential patterns of identified markers between various sample classes, we visualized the methylation signals on the markers across those sample classes (e.g. the RRBS data of solid tissues, and the cfMethyl-Seq and WGBS data^15^ of plasma cfDNA samples) with unsupervised clustering heatmaps (Supplementary Fig. S2–S4). Additional scatter plots further confirmed these markers’ consistent methylation signals between the marker discovery sample set (tumor tissues versus adjacent normal tissues) and the plasma sample set (cfDNA from cancer versus noncancer subjects) (Supplementary Fig. S5). Furthermore, we used the 450K methylation array data of the Cancer Genome Atlas^15^ (TCGA) as an independent data source to confirm the markers’ differential power between cancer and noncancer tissue samples (Supplementary Fig. S6).

Both cancer-specific and tissue-specific hypermethylation markers are enriched in promoters, 5’-UTR, simple repeats, H3K4me2, H3K4me3, and H3K9ac, while hypomethylated markers are enriched in Alu, LINE, SINE repeats, and H3K9me3. Further inspection of the MSigDB^16,17^ showed that 36% of genes adjacent to or inside the top 2000 cancer-specific markers are involved in known cancer modules, and 44% of those are part of oncogenic signatures that are activated in cancer cells upon perturbation in KRAS, PRC2, and TP53. Moreover, 68% (56%) of genes, the promoter regions of which overlap with the top 50 cancer-specific hyper- (hypo-) methylation markers of each cancer type, were reported in cancer-related publications (details in Supplementary Information S4 and Supplementary Tables S7 and S8). Intriguingly, the different types of methylation markers tend to be enriched in different nuclear locations, with increasing or decreasing trends towards the periphery, center, or mid-range of the nucleus. This hint of 3D genome structural dependence in cancer methylation patterns could warrant further investigation (details of 3D genome structure analysis in Supplementary Information S4).

### Methylation signal deconvolution and ensemble learning

Given the cfMethyl-Seq data of a patient’s cfDNA sample, we deconvolve the reads likely to be derived from a tumor or from a specific tissue, based on their methylation patterns, from all reads falling in the cancer-specific or tissue-specific marker regions, respectively (see Methods). For each of the four types of markers (hyper/hypo-methylation and cancer/tissue-specific), we construct a profile vector where the length of the vector is the number of markers and the value in each entry is the logarithm of cancer-specific (tissue-specific) read counts.

The four feature profiles have different value scales and statistical distributions, because hypermethylated cfDNAs are more protected by nucleosomes and therefore generally have higher abundances than hypomethylated cfDNAs, for example, and tissue-specific cfDNAs are generally more abundant than cancer-specific cfDNAs due to their baseline existence in blood. To maximize the diagnostic power of the features, we employed a stacked ensemble classification framework with two layers: Level-1 consists of four independent predictive models, each using only features of one type (i.e., one “view” of the samples). Level-2 “stacks” the predictions of the four level-1 models as inputs to make a final prediction (Supplementary Fig. S7). We developed two stacked ensemble models: one for detecting cancer, and another for locating cancer. For both stacked ensemble models we used the linear support vector machine at level 1, and the random forest at level 2 (details in Methods and Supplementary Information S5).

### Performance of the cancer detection and tissue-of-origin prediction

The cfMethyl-Seq data of 408 cfDNA samples were collected from 191 noncancer individuals and 217 cancer patients (49, 30, 106, 32 from colon, liver, lung, and stomach cancer patients, respectively). Note that the noncancer individuals are not restricted to healthy individuals, but also include patients of various noncancer diseases (e.g. cirrhosis, pancreatitis, hepatitis, diabetes, etc.), reflecting realistic clinical scenarios. We performed the random-split validation^7,8,18^ on the 408 cfDNA samples as follows: from the 217 cancer cfDNA samples we randomly selected 75% to be the training data and 25% to be the test data; from the 191 noncancer cfDNA samples we randomly selected 25% for the test data, and among the remaining 75%, we randomly reserved 30 noncancer cfDNA samples solely for marker discovery and used the remaining noncancer cfDNA samples as the training data (Fig. 2a). The split was conducted 10 times, and the average and 95% confidence interval (CI) of the performance metrics in the test data across the 10 runs were reported. Supplementary Fig. S8a shows the distribution of prediction scores for all 10 runs.

**Fig. 3:**
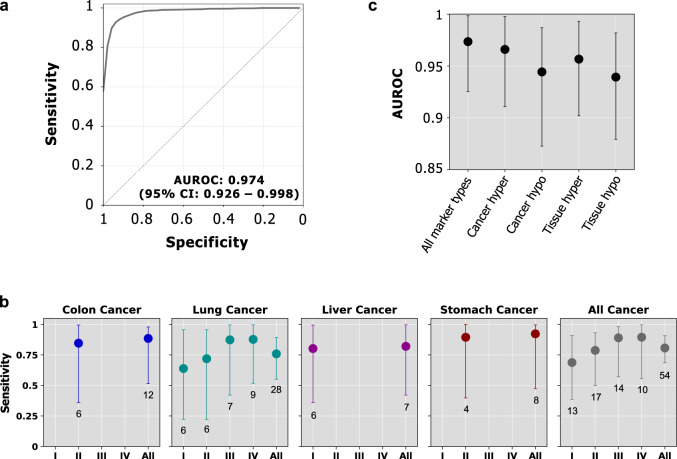
Performance of the stacked ensemble model for cancer detection. **a** ROC curve of the stacked ensemble method for detecting all four cancer types. Source data are provided as a source data file. **b** Sensitivity breakdown in each cancer stage and cancer type. Sensitivity is shown at 1 false positive (97.9% specificity). The average number of test cancer patients in each cancer type and stage over 10 runs is indicated in the label of the horizontal axis. Sensitivity is not computed if the average number of cancer patients in a cancer stage/type over 10 runs is <4. The points and error bars represent the average sensitivity over 10 runs and 95% confidence intervals. Source data are provided as a Source Data file. **c** Performance (AUROC) of using all marker types and each individual marker type ($n:$ 102 samples). The points and error bars represent the average AUROC over 10 runs and 95% confidence intervals. Source data are provided as a Source Data file.

All individual marker types achieved AUROC > 0.9 in cancer detection. The ranking of individual marker types from highest to lowest performance is cancer-specific hypermethylation markers (0.966, 95% CI: 0.911–0.998), tissue hypermethylation markers (0.957, 95% CI: 0.902–0.993), cancer-specific hypomethylation markers (0.944, 95% CI: 0.873–0.987), tissue hypomethylation markers (0.939, 95% CI: 0.879–0.982). By integrating four marker types, our cancer detection ensemble model achieved an AUROC of 0.974 (95% CI: 0.926 to 0.998), yielding a sensitivity of 80.7% (95% CI: 68.6%–90.7%) at 97.9% specificity (one incorrectly classified normal sample) (Fig. 3a). By comparing AUROCs of 10 runs between the stacked ensemble model and each of four individual marker types (i.e. the Level 1 classifiers in the stacked model), the one-sided paired Wilcoxon signed-rank test showed that the AUROC of the ensemble model is not significantly greater than that of cancer-specific hypermethylation markers (*P* value = 0.07), and significantly greater than those of the other three marker types (all *P* values <0.002). For early-stage cancer (stages I and II) samples, our ensemble model achieved an AUROC of 0.964 (95% CI: 0.897 to 0.999), with a sensitivity of 74.5% (95% CI: 54.1%–87.7%) at 97.9% specificity. When breaking down these results to individual cancer types and stages (Fig. 3b), we achieved sensitivities at or above 63% in all cases.

**Fig. 4:**
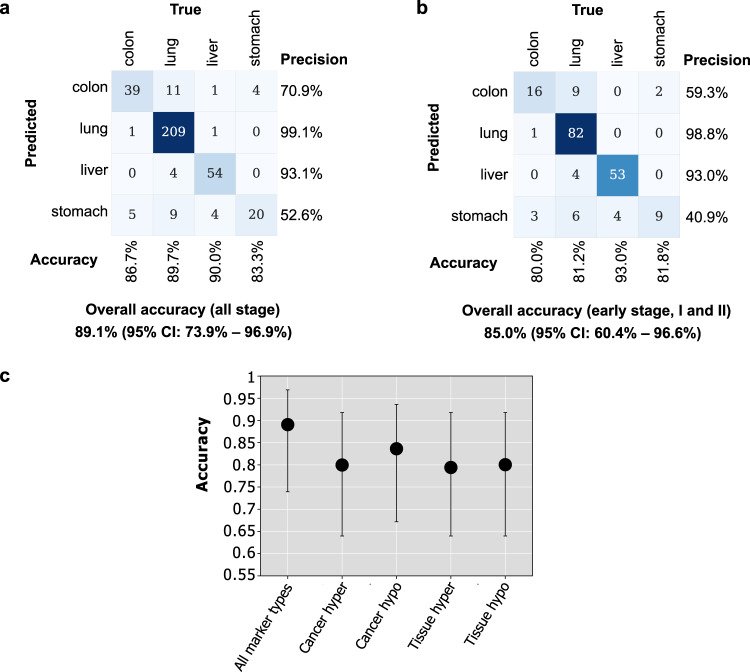
Performance of the stacked ensemble model for cancer Tissue-Of-Origin prediction. **a** Confusion matrix for all-stage cancer samples. Source data are provided as a Source Data file. **b** Confusion matrix for early-stage (i.e., stage I/II) cancer samples. Source data are provided as a Source Data file. **c** The accuracy of using all marker types and each individual marker type ($n:$ 35 samples in the test set of each run). The points and error bars represent the average accuracy over 10 runs and 95% confidence intervals. Source data are provided as a Source Data file.

We used the same validation strategy to evaluate the performance of TOO prediction on the cancer samples. Across the 10 runs and all cancer stages of 4 organ sites (colon, liver, lung, stomach), the average accuracies of all four individual marker types are 80.0% (95% CI: 64.0%–91.8%) for cancer-specific hypermethylation markers, 83.6% (95% CI: 67.2%–93.6%) for cancer-specific hypomethylation markers, 79.4% (95% CI: 64.0%–91.8%) for tissue-specific hypermethylation markers, and 80.0% (95% CI: 64.0%–91.8%) for tissue-specific hypomethylation markers. Among the four marker types, the hypomethylation markers are more predictive for TOO prediction than the hypermethylation markers. By integrating four marker types, our TOO prediction ensemble model achieved an average accuracy of 89.1% (95% CI: 73.9%–96.9%) across all stages. By comparing accuracies of 10 runs between the ensemble model and each of the four individual marker types, the one-sided paired Wilcoxon signed-rank tests showed that the AUROC of the ensemble model is significantly greater than that of the individual marker types, with *P* value = 0.001, 0.004, 0.001, and 0.004 for cancer-specific hyper-/hypo-methylation markers and tissue-specific hyper-/hypo-methylation markers, respectively. For early-stage cancer patients, our model achieved an average accuracy of 85.0% (95% CI: 60.4%–96.6%). Particularly, the TOO prediction accuracies of all-stage colon/liver/lung/stomach cancer are 86.7%/89.7%/90.0%/83.3% (Fig. 4a), and 80.0%/81.2%/93.0%/81.8% (Fig. 4b) for early-stage colon/liver/lung/stomach cancer.

### Impact of the number of markers and the training sample size on the performance

**Fig. 5:**
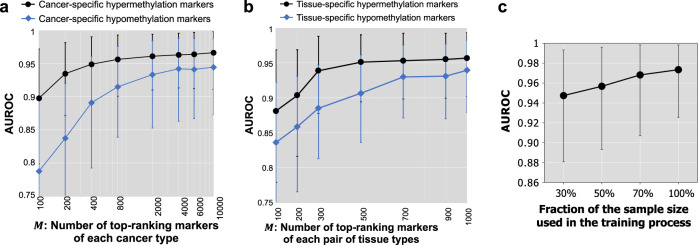
Impact of the number of markers and the training sample size on the cancer detection performance. **a** Performance of using the union of top *M* cancer-specific markers of four cancer types. Source data are provided as a Source Data file. **b** Performance of using the union of top *M* tissue-specific markers of each tissue pair. Source data are provided as a Source Data file. **c** Performance of the ensemble model for cancer detection increases with increasing training sample size (using 30% to 100% of the training samples). Source data are provided as a Source Data file. In all figures, the points and error bars represent the average AUROC over 10 runs and 95% confidence intervals ($n:$ 102 test samples per run).

We examine how the number of methylation markers impacts the performance of our classifiers. As shown in Figs. 5a, b, for all marker types the performance (AUROC) of cancer detection increases with the number of markers used. With 200 cancer-specific hypermethylation markers from each cancer type, we can already reach an AUROC of 0.935. The increasing trend of AUROC slows down after the marker number reaches 2000 (AUROC = 0.961), and the AUROC plateaus when the number of markers approaches 10,000 (AUROC = 0.966). Note that here we detect only four types of cancer, so if we want the predictive model to cover more types of cancer and covariates, the total number of combined markers will likely further increase. These results highlight the advantage of using the whole methylome rather than a small panel to cover the heterogenous cancer and population landscape for enhanced performance.

We further examined how the training sample size impacts the classifier performance. As shown in Fig. 5c, as we increased the size of the training samples (e.g., 30%, 50%, 70%, and 100% of the original training samples), the average performance of the cancer detection model improved substantially. This observation also holds for the level-1 model of each individual marker type. Moreover, the performance variance of all predictive models for the cancer detection task decreases. This indicates that the performance of the models will improve with larger training sizes.

### Evaluating the robustness of the classifiers against potential confounders

To evaluate whether the above performance could be inflated by potential confounders such as batches, sample sources, and ages, we performed the following additional validations (Supplementary Fig. S9):

1. Cross-batch validation, where samples in the testing and training sets were from non-overlapping batches. A batch of samples is defined as a set of samples that were built into libraries and subsequently sequenced together. Specifically, we randomly selected 3 out of the total 19 batches as the testing set and used the remaining nonoverlapping batches as the training set, splitting the data 15 times such that each batch was in at least one testing set. Note that some samples were sequenced twice and their data was combined, meaning they came from two batches, and when these samples were involved in the testing set, all samples from overlapping batches were removed in the training to avoid information leak. Therefore, on average the total number of samples used in a cross-batch validation is 331 ± 36. For each cross-batch validation, as a comparison we randomly selected the same numbers of training and testing samples with matched age-distribution and cancer types from the total 408 samples. Note that due to the reduced sample size of both training and testing sets, the validation performance from this down-sized “random-split” validation is expected to be lower than that of the original sample size. The cross-batch validation achieved an AUROC of 0.943 (95% CI: 0.822–1.000), which is comparable to an AUROC of 0.954 (95% CI: 0.863–1.000) in the random-split validation, showing that our classifier is reasonably robust against batch effects.
2. Cross-source validation, where samples in the testing and training sets were collected at different sites. Specifically, we chose samples from two major sources: those collected from UCLA, and those from a commercial vendor, in total 170 samples. Using samples from UCLA for training, and those from the commercial source for testing, we achieved an AUROC of 0.992 (95% CI: 0.970–1.000). We then randomly selected the same number of training/testing samples with matched age-distribution and cancer types from the 408 samples 10 times to conduct the random-split validation, yielding a comparable AUROC of 0.982 (95% CI: 0.862–1.000). Reversing training/testing for both validations, we achieved the AUROC of 0.954 (95% CI: 0.876–1.000) for cross-source validation, comparable to the AUROC of 0.951 (95% CI: 0.849–1.000) of the random-split validation.
3. Age-matched validation, where we performed the random-split validation but added a constraint of selecting only those noncancer samples that match the age-distribution of all cancer samples in both training and testing sets, respectively. This procedure was performed 10 times with the average sample size 310 ± 2 and their average AUROC is 0.948 (95% CI: 0.786–1.000), comparable to an AUROC of 0.931 (95% CI: 0.796–0.999) of the random-split validation by keeping the same training and testing sample sizes as those of the age-matched validation. Our classifiers’ robustness to age can possibly be attributed to the fact that we used paired tumor and adjacent normal tissues from the same patients for marker selection, therefore any marker selected should be due to tumor/normal differences, not age differences.
4. Independent validation, where we used our data (generated with the cfMethyl-Seq platform) as the training set, and applied the level-1 classifier of cancer detection to a published WGBS dataset of 66 plasma samples (including 40 noncancer and 26 liver cancer samples)^19^. Specifically, for each sample in the independent validation set, its prediction score is averaged across the 10 scores assigned by the 10 classifiers, each trained by a random split of cfMethyl-Seq data, and the AUROC was generated based on the average prediction scores of all samples of the independent validation set^2^. For the level-1 models, we achieved an AUROC of 0.998 (95% CI: 0.991–1.000) and 0.956 (95% CI: 0.899–0.989) using cancer-specific hypomethylation and hypermethylation markers, respectively, comparable to the published cross-validation performance within the same WGBS dataset^7,19^, demonstrating the robustness of our markers and level-1 classifiers. Tissue-specific hypomethylation and hypermethylation markers yielded lower performance at the AUROCs of 0.939 (95% CI: 0.880–0.985) and 0.882 (95% CI: 0.791–0.957), respectively. This is reasonable, since the independent WGBS data has very low coverage (1x~3x), which is a challenging scenario for revealing subtle tissue-specific signals, particularly for early-stage cancer patients, due to the existing tissue baseline signals and their variances. The level-2 random forest classifier, learned from the cfMethyl-Seq platform, however, cannot be directly applied to the WGBS platform, because the level-1 output from different platforms (cfMethyl-Seq and WGBS) have different ranges (see Supplementary Fig. S10a, b), demanding different branching cutoff values in the level-2 random forest model (see an example in Supplementary Fig. S10c). Therefore, a direct validation of our level-2 random forest classifier is not appropriate with this WGBS dataset. Nevertheless, to validate the “spirit” of the stacked level-2 feature integration, instead of random forest we trained linear classifiers (such as logistic regression and linear SVM) on the cfMethyl-Seq data and then applied the trained classifier to the WGBS data. Because the AUROCs of linear models depend only on the relative ranks of the prediction scores among samples, linear models are more interoperable between different platforms than random forest models that use absolute branching cutoffs. Note that due to the insufficient sequencing coverage for tissue-specific markers, we only used the level-1 outputs of cancer-specific hypermethylation and hypomethylation markers as the input for the level-2 linear classifiers, which achieved AUROCs of 0.998 (95% CI: 0.990–1.000 for logistic regression; and 95% CI: 0.991–1.000 for linear SVM), showing the dominant contribution from the level-1 cancer-specific hypomethylation markers (Supplementary Fig. S10d). Supplementary Fig. S8b shows the distribution of the average prediction scores for level-1 and level-2 classifiers.

The above four validations demonstrated that our cancer detection classifiers are robust against potential batch effects, different samples sources, age, and even different experimental platforms (for level-1 classifiers). For cancer TOO prediction, there are not sufficient numbers of samples to perform analyses to support the above validations.

## Discussion

We develop an integrated experimental and computational system to address the major challenges of cfDNA-based early cancer detection: i.e., the low tumor burden in blood, the molecular heterogeneity of cancer, and the fact that currently available training sample sizes are too small to accurately represent the heterogeneity of the disease and the patient population.

Our experimental assay, cfMethyl-Seq, cost-effectively profiles the genome-wide methylation of cfDNA, offering >12× enrichment over WGBS in CpG islands. A recent approach, cf-RRBS^20^, has also attempted to cost-effectively profile the cfDNA methylome. The cf-RRBS method blocks input cfDNA fragments only on their 5’-ends. In contrast, our cfMethyl-Seq procedure blocks the input cfDNA fragments on both ends. Thus, cfMethyl-Seq prevents all input cfDNA fragments lacking the desired digestion sites from ligating to adapters. In cf-RRBS, cfDNA fragments without the desired digestion sites can still ligate to adapters, but this process forms a nick between the adapter and the cfDNA fragment. A subsequent step removes the nicked fragments with exonuclease digestion^21^. However, exonuclease digestion removes all DNA fragments that contain nicks, a scenario that has been estimated to occur in 30% of cfDNA fragments^22^, leading to the loss of precious cfDNA. Hence, we expect that the cfMethyl-Seq library retains significantly more cfDNA. Furthermore, in cf-RRBS, adapters and ligases are consumed by the majority of cfDNA fragments, including those without the desired digestion sites, leading to lower ligation efficiency for the fragments of interest and lower library yields. Another cfDNA methylome sequencing method, cfMeDIP^23^, was primarily developed to bypass bisulfite conversion and allow for low input amounts. cfMeDIP uses immunoprecipitation to pull down cfDNA fragments with at least one methylated CpG site. This standard, however, applies to ~50% of all cfDNA fragments. Further refining wash conditions may help to enrich the CpG-rich fragments, although this still remains to be demonstrated. Note that cfMeDIP does not have base-pair resolution, so it cannot determine which specific CpG sites in a cfDNA fragment are methylated. Other existing approaches using targeted methylation^2^ need to establish fixed panels a priori, so their data cannot be used to discover new markers as sample sizes increase.

From cfMethyl-Seq profiles, we extract four types of cfDNA methylation information in blood. We demonstrate the power of cfMethyl-Seq in a cohort of 217 colon, liver, lung, and gastric cancer patients and 191 individuals without cancer, for detecting cancer and locating cancer. For cancer detection, we achieved a sensitivity of 80.7% (95% CI: 68.6%–90.7%) across all stages and 74.5% (95% CI: 54.1%–87.7%) for stages I and II, with a specificity of 97.9%. For locating a tumor’s TOO, we achieved an accuracy of 89.1% (95% CI: 73.9%–96.9%) for all stages and 85.0% (95% CI: 60.4%–96.6%) for early stages. While the cancer hypomethylation markers are most predictive for TOO and integrating other marker types further significantly improve the TOO prediction, the cancer hypermethylation markers are most informative for cancer detection and integrating more marker types does not improve the cancer detection performance significantly. To further confirm that our classifiers are robust to various potential confounders, we have performed extensive validations, e.g. cross-batch, cross-sample-source, age-matched, and independent validation (data generated with different experimental platforms and in different laboratories). We believe that the robustness of the classifiers can be at least partially attributed to our identification of tumor methylation signals at the read-level, where individual reads are assessed in terms of their likelihood to come from tumors.

Finally, our data show that as training sample sizes increase, the detection power of our method continues to increase. Although all existing cancer detection studies are limited by training sample sizes, cfMethyl-Seq uniquely and cost-effectively retains the genome-wide epigenetic profiles of cancer abnormalities, thereby permitting the classification models to learn and exploit newly significant features as training cohorts grow, as well as expanding their scope to other cancer types. Therefore, cfMethyl-Seq can truly facilitate a big data approach for cancer detection.

## Methods

The institutional review board (IRB) of the University of California at Los Angeles approved this study (IRB#19-000618, IRB#19-000230, IRB#19-001488, IRB#16-000659, IRB#17-000985), and our research complies with all relevant ethical regulations. All participants gave their written informed consent.

### Plasma and solid tissue sample source

Plasma samples of cancer patients were collected at UCLA hospitals (75 from Ronald Reagan UCLA Medical Center and UCLA Santa Monica hospital) or purchased from Biopartners, Inc. (Woodland Hills, CA) (200 samples). Plasma samples of patients without cancer were collected at UCLA hospitals^24^ (86 samples) or purchased from Biochain Institute, Inc (Newark, CA) (49 samples) or Biopartners, Inc. (69 samples). The solid tumor tissue and adjacent normal samples were collected from the UCLA Translational Pathology Core Laboratory (44 pairs), the UCLA Lung Cancer SPORE Bank (2 pairs), or purchased from Origene, Inc. (Rockville, MD) (37 pairs), Biochain Institute, Inc (10 pairs), Gundersen, Inc (La Crosse, WI) (4 pairs), or Biopartners, Inc. (4 pairs). The enrollment criteria at UCLA hospitals are as follows: (1) at least 18 years old, (2) able to give consent, and (3) either patients without cancer or diagnosed as colon cancer, liver cancer, lung cancer, or gastric cancer. The enrollment criteria for commercial sources are as follows: (1) at least 18 years old, (2) either patients without cancer or diagnosed as colon cancer (adenocarcinoma), liver cancer (hepatocellular carcinoma), lung cancer (squamous cell or adenocarcinoma), or gastric cancer (adenocarcinoma) (3) known stage, age, gender, and grade, and (4) no cancer treatment at time of collection. However, the sources of solid tissue samples were not required to have no cancer treatment. The characteristics of the patients who provided plasma samples are in Supplementary Table S9. The characteristics of the patients who provided tissue samples are listed in Supplementary Table S10. The amount of starting plasma is 1–4 ml. cfDNA was extracted from plasma samples with QIAGEN QIAamp circulating nucleic acid kits (Catalog# 55114, Germantown, MD) following their protocol. The solid tissue gDNA samples were extracted with QIAGEN blood and tissue kits (Catalog# 69506). 10–100 mg of tissue was used to extract gDNA from each sample.

### cfMethyl-Seq library construction

5–40 ng of cfDNA in the volume of 25 µl was used as input material. 5’-end dephosphorylation was done with 3 µl 10xCutSmart buffer and 2 µl quick CIP from NEB (Ipswich, MA) at 37^o^C for 30 min then heat-inactivated at 80^o^C for 5 min. The 3’-end blocking was done with 0.5 µl 10xCutSmart buffer, 3 µl 2.5 mM CoCl_2_, 1 µl terminal transferase (all from NEB) and 0.5 µl 1 mM ddGTP at 37 ^o^C for 2 h followed by 75 ^o^C for 20 min. The mixture was then purified with 2x AmpureXP beads (Beckman Coulter, Indianapolis, IN) and eluted in 21.5 µl RT-PCR grade water (Thermo-Fisher, Waltham, MA). Restriction digestion was done with 2.5 µl 10xCutSmart buffer and 1 µl MspI (NEB) for 18 h at 37 ^o^C and 20 min at 65^o^C. 0.5 µl 10xCutSmart buffer, 0.3 µl dACGTP mixture (100 mM dATP, 10 mM dCTP, 10 mM dGTP), 1 µl Klenow (exo-, 5U/µl, NEB) and 2.6 µl RT-PCR water, 0.6 µl 50 mM DTT (ThermoFisher) was added to the mixture for end repair and A-overhang addition with the program 30 ^o^C for 20 min, 37 ^o^C for 1 h and 75 ^o^C for 20 min. Adapter ligation was then performed with 1 µl 10xThermoFisher HC T4 ligase buffer, 0.4 µl 100 mM ATP (ThermoFisher), 0.2 µl 50 mM DTT, 1 µl ThermoFisher HC T4 DNA ligase (30 Weiss Unit/µl), 5 ng home-made duplex UMI adapter with all the cytosines methylated (protocol adopted from Kennedy et al^25^.) at 16 ^o^C for 20 h and 65 ^o^C for 20 min. Bisulfite conversion of the adapter-ligated product was carried out with QIAGEN EpiTect plus DNA bisulfite kit following their protocol for two rounds of conversion. The converted product was purified with Qiagen MinElute spin column and eluted with 20 µl RT-PCR water. PCR amplification was done using the NEBNext Multiplex Oligos for Illumina (2.5 µl of universal and index primer each) and 25 µl KAPA HiFi HotStart Uracil+ ReadyMix (Roche) with the following cycling conditions: 98 ^o^C for 45 s, 15 cycles of 98 ^o^C for 15 s, 60 ^o^C for 30 s and 72 ^o^C for 30 s, followed by a final extension at 72 ^o^C for 5 min. The PCR product was purified with 1x AmpureXP beads and eluted with 30 µl EB buffer. DNA concentration measured by Qubit 1xdsDNA HS assay, 5% TBE-UREA PAGE and bioanalyzer assay was performed as quality control on each library before sequencing. Sequences of oligos used in this study are provided in Supplementary Table S11.

### Sequencing setting, data preprocessing

cfMethyl-Seq libraries were generated for 479 cfDNA samples and were sequenced with 150 bp paired-end reads on Illumina machines by Genewiz, Inc. (South Plainfield, NJ, USA). Among them, 408 samples passing the quality control criteria (coverage higher than 15x and bisulfite conversion rate higher than 98.7%) were used in this study. We performed three steps to preprocess the cfMethyl-Seq data. In Step 1, the UMI sequence was removed and the read was trimmed. Our custom adapters contain an 8 bp random UMI and a 5 bp fixed sequence at the beginnings of both forward and reverse reads. These sequences are removed before adapter trimming (and written into the read name). Then Trim-galore^26^ was used to trim the default Illumina adapters from the sequencing reads (using the options --three_prime_clip_R1 15 --three_prime_clip_R2 13 --clip_R2 2 --length 15 --phred33). In Step 2, we performed sequence alignment, deduplication and methylation calling. We first used Bismark^11^ to align the trimmed reads to the reference genome hg19 (GRCh37 (GCA 000001405.1)). Then Umi-Grinder^27^ was used to remove PCR duplicates based on the UMI labels (now in the read names), allowing 4 mismatches in the total 16 bp UMI. Bismark^11^ methylation extractor was then used to call methylation in the mapped, deduplicated reads. In Step 3, the chromosome-wise sequence alignment statistics and whole-methylome methylation statistics of CpG islands, CpG shores, gene promoters and repetitive regions were summarized from the individual read information obtained in Step 2. Summary statistics of the cfMethyl-Seq data are available in Supplementary Table S12 with column explanations in Supplementary Table S13. In Step 4, the mapping locations of R1 and R2 were merged to form one fragment. Tissue RRBS samples were sequenced and processed in the same manner as cfMethyl-Seq data.

### Read-based discovery of methylation markers

Given the heterogeneity of DNA extracted from tumor tissues or plasma, we developed a marker discovery framework to stratify tumor-like DNA fragments from background fragments in order to sensitively capture tumor signals. Conventional methods for methylation marker discovery rely on population-average methylation values, the *β*-value, defined as the number of methylated alleles out of all alleles mapped to a CpG site in a DNA sample. Such *β*-value-based approaches, however, are not sensitive to tumor signals if the tumor fraction is low in the sample. We previously proposed the concept of *α*-value^7^, defined as the percent of methylated CpGs out of all CpG sites in a sequencing read. Supplementary Fig. S11a shows a conceptual illustration of *α*-value and *β*-value. The *α*-value of an individual sequencing read captures the pervasive nature of methylation, therefore allowing us to “purify” tumor DNA reads from background reads for enhancing the signal-to-noise ratio. A recent study further used the *α*-value concept for identifying liver cancer methylation markers^28^. Here we developed a general framework for using read-level *α*-values to robustly identify markers from impure tissue samples or even cfDNA plasma samples. The essence of our marker discovery method is to (1) compare the samples at the read level, by the *α*-values of individual sequencing reads in a genomic region (so called “*α*-value distribution” for each sample, see Supplementary Fig. S11b); and (2) find regions where the *α*-value distributions of the sequencing reads in positive samples have a well-separated component from those in the negative samples. Since *α*-value distributions often do not follow any known statistical distributions, we develop a non-parametric method to compare two *α*-value distributions. Taking hypermethylation marker discovery as an example, our marker discovery method automatically determines an *α*-value threshold, i.e., ${\alpha }_{{{{{{\rm{hyper}}}}}}}$, where reads with *α*-values $\ge {\alpha }_{{{{{{\rm{hyper}}}}}}}$ are defined as hypermethylated reads. Given an ${\alpha }_{{{{{{\rm{hyper}}}}}}}$, if hypermethylated read counts of tumors are significantly larger than those of their adjacent normal tissues, then this genomic region carries significant tumor signals (Supplementary Fig. S11c). The more pairs of tumor and adjacent normal tissues that demonstrate such tumor signals, the more stable the marker is. The same principle applies to the hypomethylation marker discovery, where an *α*-value threshold, i.e., ${\alpha }_{{{{{{\rm{hypo}}}}}}}$, will be determined and those reads with *α*-values $\le {\alpha }_{{{{{{\rm{hypo}}}}}}}$ are defined as hypomethylated reads. The framework can be flexibly adapted to (1) identify the hyper- or hypo-methylation markers; (2) discover markers from tumor tissues (with or without adjacent normal tissues), plasma samples, or normal tissues (for tissue-specific markers); (3) discover markers for cancer detection and TOO prediction. Refer to Supplementary Information S1–S3 for a detailed description.

### Generation of the methylation marker profiles with the read-based signals

Using the read-based methylation marker discovery method, each marker is associated with two thresholds which are learned from the data, i.e., ${\alpha }_{{{{{{\rm{hypo}}}}}}}$ and ${\alpha }_{{{{{{\rm{hyper}}}}}}}$, which define hypomethylated and hypermethylated reads, respectively. Therefore, for hypermethylation markers, we can identify those reads whose $\alpha$-values $\ge {\alpha }_{{{{{{\rm{hyper}}}}}}}$ as cancer-specific or tissue-specific hypermethylated reads, normalize the number of these reads by the sample sequencing depth, and use a logarithm transformation as the final input profile value of this marker, i.e., $\widehat{{{{{{\rm{count}}}}}}}\left({{{{{\rm{marker}}}}}}\right)={{{{{\rm{ln}}}}}}\left({10}^{9}\frac{{{{{{\rm{count}}}}}}({{{{{\rm{marker}}}}}})}{{{{{{\rm{raw}}}}}\; {{{{{\rm{read}}}}}}\; {{{{{\rm{count}}}}}}\; {{{{{\rm{of}}}}}}\; {{{{{\rm{genome}}}}}}}}+1 \right)$. Similarly, for hypomethylation markers, we can identify those reads whose $\alpha$-values $\le {\alpha }_{{{{{{\rm{hypo}}}}}}}$ as cancer-specific or tissue-specific hypomethylated reads, normalize the number of these reads by the sample sequencing depth, and use a logarithm transformation as the final input profile value of this marker. The normalized, logarithmic read counts of all markers are concatenated into a vector, which is used as the methylation profile of the cfDNA sample.

### Ensemble predictive model integrating multiple methylation marker types

The conceptual illustration of the stacked ensemble learning model for cancer detection and TOO prediction is shown as a two-level structure in Supplementary Fig. S7 and the details of training the stacked ensemble model and making predictions are shown in Supplementary Fig. S12. For both cancer detection and TOO prediction, in level 1, a Linear Support Vector Machine (LSVM) classifier (with the L2 penalty) is trained for each of the four feature profiles generated from (1) tumor-derived read counts of cancer-specific hypermethylation markers, (2) tumor-derived read counts of cancer-specific hypomethylation markers, (3) tissue-derived counts of tissue-specific hypermethylation markers, (4) tissue-derived counts of tissue-specific hypomethylation markers. We set the $C$ parameter to $C=1$ and all other hyperparameters use the default values provided by the python scikit-learn machine learning package^29^. In level 2, a random forest model with 2000 trees and the default values provided by the python scikit-learn machine learning package^29^ for all other hyperparameters are used to make the final prediction.

We implemented ensemble models separately for cancer detection and TOO prediction:

1. Cancer detection model. The output prediction score of the ensemble model is the probability of getting cancer. When the prediction score is less than a threshold, the subject is predicted as noncancer, otherwise the subject is predicted as cancer.
2. Cancer TOO prediction model. Here, one-vs-rest multiclass classifiers are used for both level-1 and level-2 models. We performed cancer TOO prediction for all cancer samples. The plasma cfDNA samples of cancer patients are predicted to be from one of four classes: colon, liver, lung, and stomach cancer. The prediction score of the ensemble model is the cancer-type-membership probability for each cancer type; the cancer type with the highest membership probability is the predicted cancer type. However, when two or more cancer types receive similarly high membership probabilities, we used the fold change between the highest membership probability and the second highest membership probability as a metric to indicate the cancer TOO prediction confidence. The higher this confidence is, the more certain we are in the cancer type prediction. No TOO prediction was assigned for those subjects whose cancer TOO confidence was less than a threshold. The threshold of cancer TOO confidence was set as 2.5.

### Performance evaluation of two ensemble models

For the binary classification of cancer detection, the AUROC (Area Under the Receiver Operating Characteristic curve) and the sensitivity at a certain specificity are the most popular performance metrics. For the multiclass cancer TOO prediction, the overall accuracy, i.e., ${{{{{\rm{accuracy}}}}}}=\frac{\#{{{{{\rm{correctly}}}}}}\; {{{{{\rm{predicted}}}}}}\; {{{{{\rm{samples}}}}}}}{{{{{{\rm{total}}}}}}\;\#\; {{{{{\rm{samples}}}}}}}$ is the most commonly used measure. We use a confusion matrix to further break down the overall accuracy into specific cancer TOOs for the correctly and incorrectly predicted samples. Using the confusion matrix, we can also calculate the precision for each cancer type, defined as ${{{{{\rm{precision}}}}}}({{{{{\rm{cancer}}}}}}\; {{{{{\rm{type}}}}}})=\frac{\#{{{{{\rm{samples}}}}}}\; {{{{{\rm{correctly}}}}}}\; {{{{{\rm{predicted}}}}}}\; {{{{{\rm{as}}}}}}\; {{{{{\rm{this}}}}}}\; {{{{{\rm{cancer}}}}}}\; {{{{{\rm{type}}}}}}}{{{{{{\rm{total}}}}}}\;\#\; {{{{{\rm{samples}}}}}}\; {{{{{\rm{predicted}}}}}}\; {{{{{\rm{as}}}}}}\; {{{{{\rm{this}}}}}}\; {{{{{\rm{cancer}}}}}}\; {{{{{\rm{type}}}}}}}$. Due to the limited sample size, here we generated the confusion matrix by accumulating the confusion matrices calculated from over 10 runs.

### Reporting summary

Further information on research design is available in the Nature Research Reporting Summary linked to this article.

### Supplementary information

Supplementary Information

Description of Additional Supplementary Files

Supplementary Data 1

Supplementary Data 2

Supplementary Data 3

Supplementary Data 4

Supplementary Data 5

Supplementary Data 6

Supplementary Data 7

Supplementary Data 8

Supplementary Data 9

Supplementary Data 10

Supplementary Data 11

Supplementary Data 12

Supplementary Data 13

Reporting Summary

### Source data

Source Data

## Data Availability

Raw sequencing data generated in this study (RRBS data from 328 solid tissue samples and cfMethyl-Seq data from 479 plasma samples) have been deposited into the European Genome-Phenome Archive under accession code EGAS00001006020. Data access can be obtained through a request to the corresponding authors. Access to the data will be restricted to non-commercial entities. The corresponding authors will generally respond to requests within three days. Once granted, the access has no time restriction. Data from the study^19^ EGAS00001000566, (40 non-cancer and 26 liver cancer WGBS samples) were used for independent validation and are associated with Supplementary Fig. S4, S8b and S10. TCGA 450k array data were obtained from https://cancergenome.nih.gov/and are associated with Supplementary Fig. S6. Reference genome hg19 (GRCh37 (GCA 000001405.1) was used for mapping samples. The remaining data are available within the Article, Supplementary Information, or Source data. Source data are provided with this paper.

## Code availability

Software code is available at https://github.com/jasminezhoulab/cfMethyl-Seq and can be freely used for educational and research purposes by non-profit institutions and U.S. government agencies. For information on the use for a commercial purpose or by a commercial or for-profit entity, please contact Professor Xianghong Jasmine Zhou.
