# Author Correction: Cost-effective methylome sequencing of cell-free DNA for accurately detecting and locating cancer

**DOI:** 10.1038/s41467-024-48018-5

**Published:** 2024-05-01

**Authors:** Mary L. Stackpole, Weihua Zeng, Shuo Li, Chun-Chi Liu, Yonggang Zhou, Shanshan He, Angela Yeh, Ziye Wang, Fengzhu Sun, Qingjiao Li, Zuyang Yuan, Asli Yildirim, Pin-Jung Chen, Paul Winograd, Benjamin Tran, Yi-Te Lee, Paul Shize Li, Zorawar Noor, Megumi Yokomizo, Preeti Ahuja, Yazhen Zhu, Hsian-Rong Tseng, James S. Tomlinson, Edward Garon, Samuel French, Clara E. Magyar, Sarah Dry, Clara Lajonchere, Daniel Geschwind, Gina Choi, Sammy Saab, Frank Alber, Wing Hung Wong, Steven M. Dubinett, Denise R. Aberle, Vatche Agopian, Steven-Huy B. Han, Xiaohui Ni, Wenyuan Li, Xianghong Jasmine Zhou

**Affiliations:** 1 Department of Pathology and Laboratory Medicine, David Geffen School of Medicine, University of California at Los Angeles, Los Angeles, CA 90095 USA; 2 EarlyDiagnostics, Inc., 570 Westwood Plaza, Los Angeles, CA 90095 USA; 3 Department of Quantitative and Computational Biology, University of Southern California, Los Angeles, CA 90089 USA; 4 The Eighth Affiliated Hospital, Sun Yat-Sen University, Shenzhen, China; 5 Department of Microbiology, Immunology and Molecular Genetics, University of California at Los Angeles, Los Angeles, CA 90095 USA; 6 Department of Surgery, University of California at Los Angeles, Los Angeles, CA 90095 USA; 7 Department of Molecular and Medical Pharmacology, David Geffen School of Medicine, University of California at Los Angeles, Los Angeles, CA 90095 USA; 8 Westlake High School, 100N Lakeview Cyn Road, Westlake Village, CA 91362 USA; 9 Department of Medicine, David Geffen School of Medicine, University of California at Los Angeles, Los Angeles, CA 90095 USA; 10 Department of Radiological Sciences, David Geffen School of Medicine, University of California at Los Angeles, Los Angeles, CA 90095 USA; 11 Jonsson Comprehensive Cancer Center, University of California at Los Angeles, Los Angeles, CA 90095 USA; 12 VA Greater Los Angeles Health Care System, Los Angeles, CA 90073 USA; 13 Institute for Precision Health, University of California at Los Angeles, Los Angeles, CA 90095 USA; 14 Institute for Quantitative and Computational Biosciences, University of California at Los Angeles, Los Angeles, CA 90095 USA; 15 Department of Statistics, Stanford University, Stanford, CA 94305 USA; 16 Department of Biomedical Data Science, Stanford University, Stanford, CA 94305 USA

**Keywords:** Cancer screening, Software, Computational biology and bioinformatics, Statistical methods, Diagnostic markers

Correction to: *Nature Communications* 10.1038/s41467-022-32995-6, published online 29 September 2022

The original version of this Article omitted the Competing Interests below:

‘X.J.Z. and W.H.W. are board members for EarlyDiagnostics, Inc. X.J.Z. has an executive leadership position at EarlyDiagnostics, Inc.’ and

‘X.J.Z., W.L., W.H.W., and F.A. are stockholders of EarlyDiagnostics, Inc. M.L.S., W.Z., S.L., C.-C.L., Y.Zhou, Q.L., X.N. have stock options with EarlyDiagnostics, Inc’.

The statement: ‘The authors have filed a patent application on methods described in this manuscript’

has been replaced with the statement:

‘X.J.Z., M.L.S., Y.Zhou, X.N., and W.Z. are inventors on a patent application submitted by the Regents of the University of California and licensed to EarlyDiagnostics, Inc. (Patent No. US20210404007A1). P.S.L. performed summer internships in EarlyDiagnostics, Inc. in 2021 and 2022’.

in the corrected article.

In addition, the original Competing Interests section contained the incorrect author initials ‘S.L.’, instead of ‘W.L’.

This has been corrected in both the PDF and HTML versions of the Article.

