# Supplementary material for: Cost-effective methylome sequencing of cell-free DNA for accurately detecting and locating cancer
Source: Nat Commun. 2022 Sep 29;13:5566. doi: 10.1038/s41467-022-32995-6 (PMC9522828; doi:10.1038/s41467-022-32995-6)
Supplement: Supplementary file 1 — Supplementary Information [file 41467_2022_32995_MOESM1_ESM.pdf]

Supplementary Information

Cost-effective Methylome Sequencing of Cell-free DNA for Accurately Detecting and Locating Cancer

S1. Read-based discovery of cancer-specific methylation markers for cancer detection

The read-based marker discovery framework uses α-value, defined as the percent of methylated CpGs out of all CpG sites in a sequencing read[1]. The α-value describes the pervasiveness of the methylation in a read, and the α-values of all reads from all samples in a genomic region form an α-value distribution. The principle of our marker discovery framework is to find marker regions where the α-value distributions in positive samples have a well-separated component from those in the negative samples. This evaluation can be performed using either parametric or non-parametric methods. Since such α-value distributions often do not follow any known statistical distributions, below we describe a non-parametric method to evaluate if a region is qualified to be a hypermethylation marker (hypomethylated in most negative samples and hypermethylated in at least a subset of positive samples). This method can also be applied to identify hypomethylation markers, which are hypermethylated in most negative samples and hypomethylated in at least a subset of positive samples. Although the discovery of hyper-and hypo-methylation marker discovery follow the same principle (i.e., three criteria), we describe them separately for ease of understanding.

S1.1. Discovery of cancer-specific hypermethylation markers for cancer detection: Given tumor tissues (as positive samples), 30 noncancer plasma samples (as negative samples) and adjacent normal tissues, for each candidate marker region we aim to identify two α-value thresholds, i.e., α_{hypo} and α_{hyper} (a read is defined to be hypomethylated if its $\alpha \leq \alpha_{\mathrm{hypo}}$, and a read is defined to be hypermethylated if its $\alpha \geq \alpha_{\mathrm{hyper}}$), where the two α-value thresholds shall satisfy three criteria:

(1) Controlling background noise in negative samples (i.e., noncancer plasma): In ≥80% of negative samples, ≥99% of reads are hypo-methylated, such that the majority of reads in negative samples are counted as background, in order to guarantee the high specificity of the marker;

(2) Identifying tumor signal in positive samples (i.e., tumors): A number (*K*) of positive samples have at least 30% more hyper-methylated reads compared to their adjacent normal samples, in order to guarantee the high sensitivity of the marker;

(3) α_{hyper} - α_{hypo} ≥ 0.5 and $\alpha_{\mathrm{hypo}} \in [0, 0.5]$ to ensure a good separation between background and signals.

A genomic region is qualified to be a candidate marker if we can identify the two thresholds α_{hypo} and α_{hyper} that satisfy the above criteria. *K* is used to rank the candidate markers in terms of their quality, and the top 10,000 candidates are selected as the cancer hypermethylation markers for a specific cancer type. This procedure is independently applied to colon, liver, lung and stomach cancer. Each hypermethylation marker is associated with an α_{hyper} for a cancer type. The final set of cancer hypermethylation markers is the union of the four sets of identified markers with at least 3 CpG sites. For each cancer hypermethylation marker in the final union set, if the marker is selected by only one cancer type, its associated α_{hyper} is used for extracting hypermethylated reads in the target samples; and if the marker is selected by ≥ 2 cancer types, it is associated with multiple α_{hyper} among which we will use the smallest α_{hyper} for extracting hypermethylated reads in the target samples. Note that the final methylation marker set is identified for each of 10 validation runs. Because each validation run has a different set of 30 non-cancer plasma samples reserved for marker discovery, the markers slightly differ between each validation run. Across 10 runs, we identified an average of 23,748 cancer-specific hypermethylation markers for cancer detection.

Using this framework, different markers have different α_{hypo} and α_{hyper} which define the hyper- and hypo-methylated reads, because different genomic regions have different magnitudes of background noise. Supplementary Fig. S11 illustrates this principle with one negative sample and one positive sample, and Supplementary Fig. S13 further illustrates this principle by using many samples.

S1.2. Discovery of cancer-specific hypomethylation markers for cancer detection: Given tumor tissues (as positive samples), 30 noncancer plasma samples (as negative samples) and adjacent normal tissues, for each candidate marker region we aim to identify two α-value thresholds, i.e., α_{hypo} and α_{hyper} (a read is defined to be hypo-methylated if its $\alpha \leq \alpha_{\text{hypo}}$, and a read is defined to be hypermethylated if its $\alpha \geq \alpha_{\text{hyper}}$), where the two α-value thresholds shall satisfy three criteria:

(1) Controlling negative samples (i.e. noncancer plasma): In ≥80% of negative samples, ≥99% of reads are hyper-methylated, such that majority of reads in negative samples are counted as background, in order to guarantee the high specificity of the marker;

(1) Identifying tumor signal in positive samples (i.e. tumors): A number (*K)* of positive samples have at least 30% more hypo-methylated reads compared to their adjacent normal samples, in order to guarantee the high sensitivity of the marker;

(2) α_{hyper} - α_{hypo} ≥0.5 and $\alpha_{\text{hyper}} \in [0.5, 1]$ to ensure a good separation between background and signals.

K is used to rank the markers in terms of their quality, and the top 10,000 candidates are selected as the cancer hypomethylation markers for a specific cancer type. This procedure is independently applied to colon, liver, lung and stomach cancer. Each hypomethylation marker is associated with an α_{hypo} for a cancer type. The final set of hypermethylation markers is the union of the four sets of identified markers with at least 3 CpG sites. For each cancer hypomethylation marker in the final union set, if the marker is selected by only one cancer type, its associated α_{hypo} is used for extracting hypomethylated reads in the target samples; and if the marker is selected by ≥ 2 cancer types, it is associated with multiple α_{hypo} among which we will use the largest α_{hypo} for extracting hypomethylated reads in the target samples. As a result, across 10 runs, we identified an average of 28,197 cancer-specific hypomethylation markers for cancer detection.

S2. Read-based discovery of cancer-specific methylation markers for cancer TOO prediction

The cancer-specific markers for TOO prediction are those genomic regions that significantly differ between solid tumor types as well as between solid tumors and the 30 noncancer plasma samples (randomly drawn and reserved for marker discovery in each of the 10 validation runs). We used RRBS data of 134 solid tumors for colon (20), liver (53), lung (46), and stomach (15) cancer (Fig. 2b). Although the discovery of hyper-and hypo-methylation markers follows the same principle, we describe them separately for ease of understanding.

S2.1. Discovery of cancer-specific hypermethylation markers for TOO prediction: For each pair of the four tumor types (denoted as tumor type A and tumor type B), we compared tumor type A (as positive samples) and tumor type B (as negative samples) to identify one-vs-one cancer hypermethylation markers (denoted as A+ vs B- hypermethylation markers) by using the following criteria (the B+ vs A- hypermethylation markers can be identified analogously):

(1) $\alpha_{\text{hyper}} = \alpha_{\text{hypo}}$, because in most regions the α-value distributions of two tumor types can be very similar, the requirement of $\alpha_{\text{hyper}} > \alpha_{\text{hypo}}$ would make almost all regions fail to pass this criterion;

(2) Controlling background noise in negative tissue samples: A stringent requirement is used to guarantee the high specificity of the marker, i.e., $\geq (N_{\text{negative}} - 2)$ negative samples have $\geq 99\%$ hypo-methylated reads, where N_{negative} is the total number of negative samples;

(3) Identifying tumor signal in positive tissue samples: A loose requirement is used to guarantee the high sensitivity of the signals, i.e., *K* (e.g., *K*≥4) positive samples have $\geq 10\%$ hyper-methylated reads.

(4) Filtering by noncancer plasma: Keep only those genomic regions which have $\geq 99\%$ hypo-methylated reads in ≥80% of 30 noncancer plasma samples.

K is used to rank the markers in terms of their quality, and the top 1,000 candidates are selected as the cancer hyper-methylation markers for each pairwise comparison between any two tumor types, resulting in 12 marker sets. Each hypermethylation marker is associated with an α_{hyper} for a pairwise comparison of two tumor types. The final set of cancer hyper-methylation markers is the union of the 12 sets of identified markers with at least 3 CpG sites. For each cancer hypermethylation marker in the final union set, if the marker is selected by only one pairwise comparison, its associated α_{hyper} is used for extracting hypermethylated reads in the target samples; and if the marker is selected by ≥ 2 pairwise comparisons, it is associated with multiple α_{hyper} among which we will use the smallest α_{hyper} for extracting hypermethylated reads in the target samples. Note that the final methylation marker set is identified for each of the 10 validation runs. Because for each validation run a different set of 30 non-cancer plasma samples were randomly drawn for marker discovery, the markers differ slightly between each validation run. Across 10 runs, we identified an average of 30,474 cancer-specific hypermethylation markers for cancer TOO prediction. This framework can also be applied to identify cancer-specific hypomethylation markers, as described below.

S2.2. Discovery of cancer-specific hypomethylation markers for TOO prediction: For each pair of the four tumor types (denoted as tumor type A and tumor type B), we compared tumor type A (as positive samples) and tumor type B (as negative samples) to identify one-vs-one cancer hypermethylation markers (denoted as A+ vs B- hypermethylation markers) by using the following criteria (the B+ vs A- hyper-methylation markers can be identified analogously):

(1) $\alpha_{\text{hyper}} = \alpha_{\text{hypo}}$, because in most regions the α-value distributions of two tumor types can be very similar, the requirement of $\alpha_{\text{hyper}} > \alpha_{\text{hypo}}$ would make almost all regions fail to pass this criterion;

(2) Controlling background noise in negative tissue samples: A stringent requirement is used to guarantee the high specificity of the marker, i.e., $\geq (N_{\text{negative}} - 2)$ negative samples have $\geq 99\%$ hyper-methylated reads, where N_{negative} is the total number of negative samples;

(3) Identifying tumor signal in positive tissue samples: A loose requirement is used to guarantee the high sensitivity of the signals, i.e., K (e.g., $K\geq4$) positive samples have $\geq 10\%$ hypo-methylated reads.

(4) Filtering by noncancer plasma: Keep only those genomic regions which have $\geq 99\%$ hyper-methylated reads in ≥80% of 30 noncancer plasma samples.

K is used to rank the markers in terms of their quality, and the top 1,000 candidates are selected as the cancer hypo-methylation markers for each pairwise comparison between any two tumor types, resulting in 12 marker sets. Each hypomethylation marker is associated with an α_{hypo} for a pairwise comparison of two tumor types. The final set of cancer hypo-methylation markers is the union of the 12 sets of identified markers with at least 3 CpG sites. For each cancer hypomethylation marker in the final union set, if the marker is selected by only one pairwise comparison, its associated α_{hypo} is used for extracting hypomethylated reads in the target samples; and if the marker is selected by ≥ 2 pairwise comparisons, it is associated with multiple α_{hypo} among which we will use the largest α_{hypo} for extracting hypomethylated reads in the target samples. Across 10 runs, we identified an average of 33,890 cancer-specific hypomethylation markers for cancer TOO prediction.

S3. Discovery of tissue-specific hyper- and hypo-methylation markers for cancer detection and TOO prediction

Since organs containing tumors undergo increased cell death and therefore yield an elevated quantity of cfDNA[2–4], tissue-specific cfDNA deconvolution can aid in both detecting cancer and predicting its TOO. In the tissue-specific methylation marker discovery, we used only normal tissues and didn't use any plasma samples. Therefore, the tissue-specific methylation markers are invariant between all 10 runs for both cancer detection and TOO prediction. Specifically, we used RRBS data of 194 solid normal tissues (28 colon tissues, 72 liver tissues, 68 lung tissues, and 26 stomach tissues) for tissue-specific methylation marker discovery (Fig. 2b). For each combination of the four tissue types (denoted as tissue type A and tissue type B), we compared tissue type A (as positive samples) and tissue type B (as negative samples) to identify one-vs-one tissue hyper- and hypo-methylation markers (denoted as A+ vs B- hyper- and hypo-methylation markers, respectively). The tissue-specific hyper- and hypo-methylation markers are identified in the same way as described in the discovery of cancer-specific hyper- and hypo-methylation markers for TOO prediction

(Section S2.1 and S2.2), respectively, except that the filtering step (i.e., step (4)) is ignored. The B+ vs A- methylation markers can be identified analogously. By choosing the top 1,000 candidates (1,000 hypermethylation and 1,000 hypomethylation markers) from each pairwise comparison between any two tissue types (12 pairwise comparisons in total), we identified 12 sets of hypermethylation markers and 12 sets of hypomethylation markers among four tissue types. Then the full set of tissue-specific hypermethylation (hypomethylation) markers is the union of 12 sets of identified markers with at least 3 CpG sites. For each tissue-specific hypermethylation (hypomethylation) marker in the final union set, if the marker is selected by only one pairwise comparison, its associated α_{hyper} (α_{hypo}) is used for extracting hypermethylated (hypomethylated) reads in the target samples; and if the marker is selected by ≥ 2 pairwise comparisons, it is associated with multiple α_{hyper} (α_{hypo}) among which we will use the smallest α_{hyper} (largest α_{hypo}) for extracting hypermethylated (hypomethylated) reads in the target samples. As a result, we identified 7,547 tissue-specific hypermethylation and 7,212 tissue-specific hypo-methylation markers that can differentiate between pairs of tissue types.

S4. Functional analysis methods

Enrichment of genomic features in methylation markers. We extracted 3' UTR and 5' UTR regions from the GENCODE database[5], and obtained the locations of CpG Islands, RefSeq exons/introns, promoters (3000bp upstream of TSS to TSS), and Repeat elements (Alu, LINE, Satellite, simple repeats, and SINE) from the UCSC genome browser database[6]. For cancer-specific hypermethylation and hypomethylation markers, we investigated the consensus methylation markers that were selected by all 10 validation runs, resulting in 21,858 cancer-specific hypermethylation markers and 22,617 cancer-specific hypomethylation markers. Because only tissue samples were used for identifying tissue-specific methylation markers, they are invariant between all 10 runs, consisting of 7,547 hypermethylation and 7,212 hypomethylation markers. To calculate the enrichment level of a given type of genomic feature with respect to each type of methylation marker, we performed the following analysis: **(1)** We generated 100 independent sets of *M* "pseudo-markers". A pseudo-marker is a genome region (i.e., size and location) drawn from the entire pool of regions initially identified as marker candidates. **(2)** For each type of genomic feature (e.g. Alu repeat elements), we calculated the number of cases where a genomic feature region overlaps one of the top *M* true methylation markers. **(3)** For each type of genomic feature, we also calculate the number of regions that overlap one of the *M* pseudo-markers. **(4)** For each type of genomic feature, the enrichment fold change is calculated as (# true markers that overlap with genomic feature regions) / (average # pseudo-markers that overlap with genomic feature regions), where the denominator is averaged over the 100 independent sets generated in step 1. As shown in Supplementary Fig. S14, this analysis demonstrates that hypermethylation markers are enriched within regions corresponding to CpG islands, promoters, 5'-UTR, and simple repeats, while the hypomethylation markers are enriched in Alu, LINE, and SINE repeat regions.

Enrichment of histone markers in methylation markers. Histone marker profiles are tissue-specific. We downloaded the broad histone peaks for the A549 lung cancer cells and the HepG2 liver cancer cell line from the UCSC Genome Browser[6], and we used these data to calculate the enrichment of lung and liver cancer-specific methylation markers within the histone marker regions. We calculated the enrichment fold changes for individual histone marker regions following the same procedure as described above for genomic feature regions. As shown in Supplementary Fig. S15 and S16, we find significant enrichment of both liver and lung cancer-specific markers in the histone marker regions: the hypermethylation markers are enriched in H3K4me2, H3K4me3, and H3K9ac. In contrast, the cancer-specific hypomethylation markers are enriched in H3K9me3. The tissue-specific hypomethylation markers are generally not enriched within any type of histone marker.

Enrichment analysis using MSigDB. To investigate whether the identified cancer-specific markers are enriched in cancer-related gene sets, we selected C4 (cancer modules) and C6 (oncogenic signatures) from MSigDB[7,8] to perform hypergeometric tests. Combining the top 2000 cancer-specific hypermethylation/hypomethylation markers selected from each cancer type (in total 5,723 hyper and 4,974 hypo markers), we identified 1,663 and 1,839 genes that overlapped with these cancer-specific markers. We found that 35% of these overlapped genes are involved in C4 (cancer modules), and 43% are involved in C6 (oncogenic signatures). Several examples of these oncogenic signatures follow.

1) The cancer-specific hypermethylation markers are enriched in up-regulated genes upon *KRAS* overexpression in epithelial kidney-cancer cell lines (*P*-value 4.4e-16).

2) Both hyper- and hypo-methylation markers are enriched in down-regulated genes upon mutated *TP53* in NCI-60 cancer cells (*P*-value 1.1e-16 and 1.1e-10 for hyper- and hypo-methylated markers, respectively).

3) The cancer-specific hypomethylation markers are enriched in down-regulated genes upon *KRAS* overexpression in epithelial lung and breast cancer cell lines (*P*-value 3.2e-11).

4) The cancer-specific hypomethylation markers are significantly enriched with the gene sets that are differentially expressed upon knockdown of the member gene in the Polycomb repressive complexes 2 (*PRC2*) (*P*-value 5.7e-10), indicating that the aberrant activation of *PRC2* may induce the loss of DNA methylation during oncogenesis[9].

Cancer-related genes that overlap with methylation markers. To further investigate the biological function of top-ranking markers, we selected the top 50 cancer-specific hyper- and hypo-methylation markers for each cancer type (in total 386 distinct markers), and then extracted the genes whose promoter regions (TSS: -4000 to +1000 bp) overlap with the genomic regions of these markers. It resulted in 91 genes for hypermethylation markers and 48 genes for hypomethylation markers. We used the NCBI GeneRIF (Gene Reference into Function) database[10] to annotate these overlapping genes. We selected the cancer related GeneRIF records using keywords (cancer, tumor, carcinoma, and metastasis). Supplementary Tables S7 and S8 show the GeneRIF annotation results. Among the 91 genes that are adjacent to hypermethylated markers, 62 (68%) were reported in the cancer related publications, while among the 48 genes that are adjacent to hypomethylation markers, 27 (56%) were reported in the cancer related publications.

We ranked these overlapping genes by the number of methylation markers in their promoter regions (Supplementary Tables S7 and S8). For the hypermethylation markers, 12 genes (*OSR2, LRRC4, SFRP1, PENK, TBX4, CLIP4, TSPYL5, PRDM14, LONRF2, SHE, TDRD10*, and *VSTM2B*) overlap with ≥ 3 hypermethylation markers in their promoter regions, among which 8 genes were reported in the cancer related publications in GeneRIF database. A brief summary is as follows: the #1 ranked gene *OSR2* (overlapping with 6 hypermethylated makers in the promoter region) was reported to be significantly hypermethylated in gastric cancer patients in the noninvasive detection of gastric cancer[11]; the #2 ranked gene, *LRRC4*, was reported as a glioma suppressor[12]; promoter methylation of *SFRP1* gene (the #3 ranked gene) was associated with lymph-node metastasis[13]; promoter methylation of *PENK* was associated with colorectal adenocarcinoma diagnosis[14]; *CLIP4* was reported as a potential biomarker for synchronous metastasis of clear cell renal cell carcinomas[15]; *TSPYL5* was reported to inhibit cell proliferation, migration and invasion in colorectal cancer[16]; the methylation-mediated repression of *PRDM14* contributed to apoptosis evasion in HPV-positive cancers[17].

For hypomethylation markers, 5 genes (*FSCN2, PIP5K1A, MSTO1, NME1*, and *NME1-NME2*) overlap with ≥ 3 hypomethylation markers in their promoter regions, among which 4 genes were reported in the cancer related publications in GeneRIF database. A brief summary is as follows: the #1 ranked gene, *FSCN2* (overlapping with 5 hypomethylated makers in the promoter region), was reported as a marker for cancer-associated fibroblasts in invasive lung adenocarcinoma[18]; *PIP5K1A,* the #3 ranked gene, was reported as a novel degradative substrate of *NEDD4* contributing to breast cancer cell proliferation[19]; *NME1-NME2* is the read-through transcription between the neighboring *NME1* and *NME2* genes; *NME1* status was significant for pathologically complete response in breast cancer patients[20]; both *NME1* and *NME2* may contribute to tumor-suppressive activity with plakoglobin[21]. In summary, cancer hypomethylation markers have less cancer-related literature than cancer hypermethylation markers. However, we still found that 55% of the overlapping genes in hypomethylation markers were reported in cancer-related literature.

Distribution of methylation markers in the 3D genome: The large number of identified DNA methylation markers prompted us to examine their spatial distributions in the 3D genome. We analyzed the distribution of markers in 3D grids defined by chromatin radial organization and subcompartments. Specifically, we used the recent method "genomic loci positioning by sequencing (GPSeq[22])" as a reference, which generated the first high-resolution radial genome organization of human HAP1 cells, providing distances between individual genomic regions and the nuclear lamina along the nuclear radius. The concentric nuclear layers derived can be further stratified into subcompartments identified from Hi-C data of the GM12878 cell line[23]. A1 and A2

consist of gene-rich euchromatic regions, B1 contains facultative heterochromatic regions, B2 is enriched at the nuclear lamina and at nucleolus-associated domains, and B3 is also enriched at the nuclear lamina but not at nucleolus-associated domains[23]. As shown in Supplementary Figure S17, in all five subcompartments, the percentage of hypomethylation markers out of all marker candidates increases from the nuclear periphery towards the nuclear center, while hypermethylation markers show the opposite trends in B1 and B2 subcompartments. We note that these are preliminary results, and that we used subcompartments defined by the GM cell line while the radial distribution scores are from HAP1 cells. However, it has been shown that subcompartments are largely conserved across different cell types[24]. It would be interesting to use chromatin subcompartments and radial distribution scores defined with matching cancer types to further validate the marker distribution trends observed here.

S5. The ensemble model learning process

The stacked ensemble learning model is a two-level structure, as shown in Fig. 2c. The predictions from a set of level-1 base models, each separately learned from an individual marker type, are used as input for training a level-2 ensemble model. This is a form of ensemble learning, an effective strategy to ward against overfitting and improve the regularization of the overall model. However, learning such a two-level ensemble model may run the risk of overfitting, if we use conventional training processes[25]. Therefore, we employed a sophisticated two-level learning process (Supplementary Fig. S12), to overcome the overfitting risk present in the typical training process[25]. This is implemented by splitting all training samples into 10 non-overlapping folds (i.e. partitions) and training the level-1 base model for each marker type on the samples in 9 folds and making predictions on the samples in the remaining 1 fold. This training and prediction process is repeated 10 times, each time using a different left-out fold. These predictions, obtained by iterating 9-fold and 1-fold of the training samples, are called “out-of-fold predictions” (OOFPs)[25,26], indicating a special way of using training samples to generate the prediction scores by themselves. The OOFPs from all 10 folds are then concatenated to form the new features for all training samples so they can be used as the training data for the level-2 meta-model. To generate the testing data of level 2, we re-train each level-1 base model for each marker type using all training samples (i.e., all 10 folds), and make predictions on the test samples. These predictions then serve as the testing data for the Level 2 meta-model.

Specifically, given a set of training samples and a set of test samples, the learning process of a two-level stacked model includes two steps, which is illustrated in Supplementary Fig. S12.

- **(Step 1)** Generation of the training data for level-2 model. All training samples are split into 10 equal-size folds. We use the samples of 9 folds to train a level-1 model and then use the trained level-1 model to generate prediction scores of the training samples in the left-out fold. We repeat this procedure for each left-out fold, until every training sample can receive the prediction score from a level-1 model. Suppose we have $K=4$ marker types, each training sample shall have K prediction scores, forming a vector profile that is used as the input training data for level-2 model. Note that (1) this step does not use any test samples and therefore no information leakage occurs; (2) all prediction scores obtained in this step are called “out-of-fold predictions” (OOFPs) in the literature[25,26], indicating a special way of using training samples to generate the prediction scores by themselves.
- **(Step 2)** Generation of testing data for level-2 model. We use all training samples to train a level-1 model and then use this trained level-1 model to generate prediction scores of the test samples. Given K marker types, each test sample shall have K prediction scores, forming a vector profile that is used as the input test data for level-2 model.

After these two steps, we then use the training data generated in step (a) to train a level-2 model and use the trained level-2 model and the testing data generated in step (b) to make the final prediction for each test sample.

Please note that (1) the use of 10-fold splits is not for evaluating the performance of any models, but just for generating the training data of level-2 model. (2) We do not tune the hyperparameters of either level-1 and level-2 models, because (i) all level-1 and level-2 models are low-complexity classifiers (i.e., linear SVM and random forest) whose default hyperparameter values (provided by the popular machine learning python package “scikit-learn”) are already sufficient to achieve high performance; and (ii) our results have shown

that different hyperparameter values of linear SVM and random forest do not lead to substantial changes in performance for our data.

S6. Supplementary figures

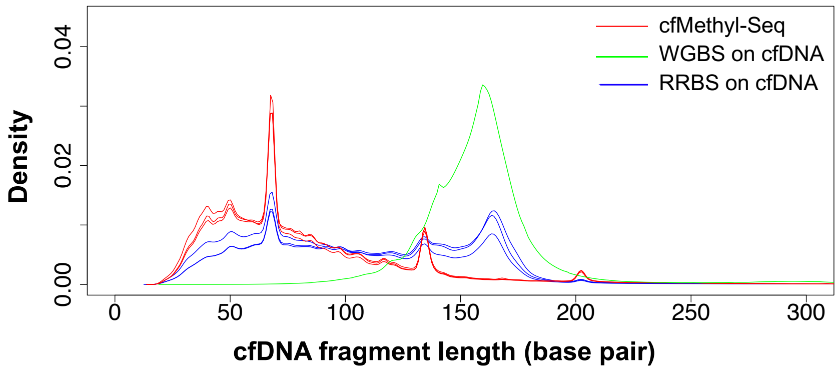

Supplementary Figure S1. The fragment length profiles of libraries sequenced with the cfMethyl-Seq protocol on cfDNA (red), compared to WGBS on cfDNA (green) and RRBS on cfDNA (blue). Source data are provided as a Source Data file.

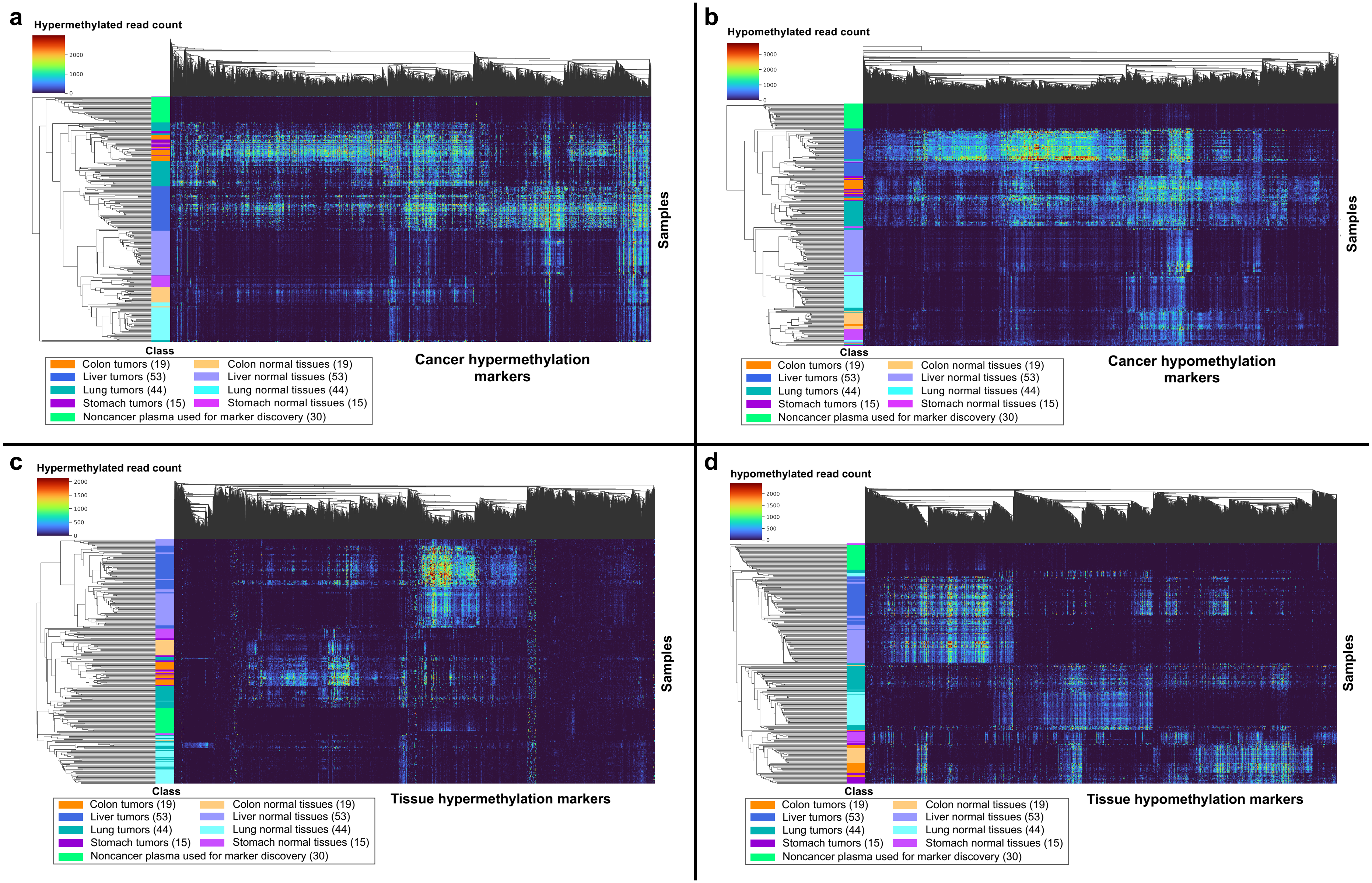

Supplementary Figure S2. Heatmaps of four types of markers, shown using the samples that are used for marker discovery (including all tumors and their adjacent normal tissues, and noncancer plasma samples used for marker discovery). **(a)** all cancer hypermethylation markers, **(b)** all cancer hypomethylation markers, **(c)** all tissue hypermethylation markers, and **(d)** all tissue hypomethylation markers. Note that hypermethylated (hypermethylated) read count refers to the count scaled by the sample sequencing depth, i.e., $\text{normalized hypermethylated read count in a marker for a sample} = 10^9 \frac{\text{Number of hypermethylated (hypomethylated) reads in a marker}}{\text{Number of reads in the sample}}$, whose unit is count per billion. Source data are provided as a Source Data file.

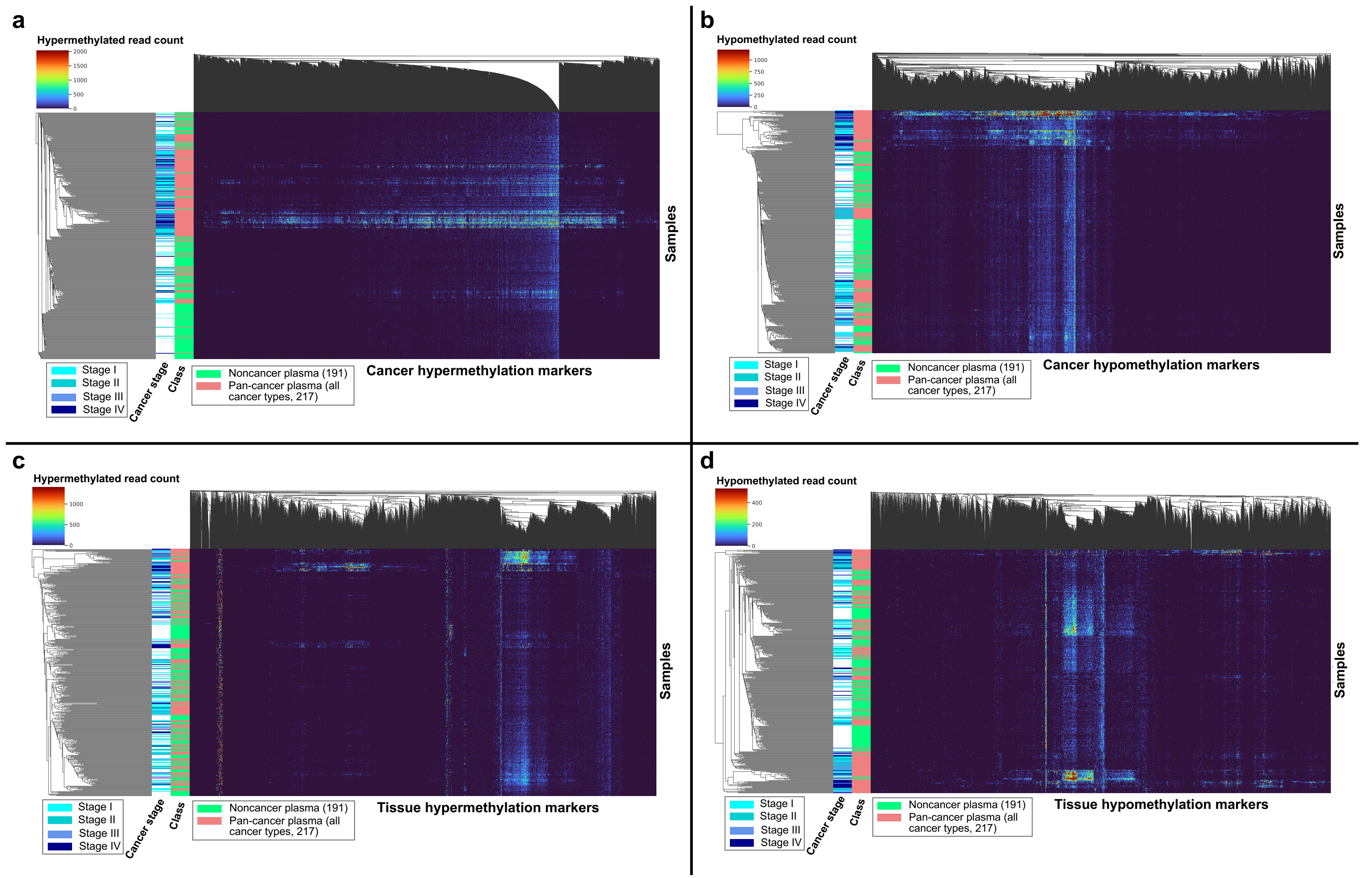

Supplementary Figure S3. Heatmaps of four types of markers shown using all plasma samples of **cfMethyl-Seq data** (including noncancer plasma samples and cancer plasma samples of all four cancer types). **(a)** all cancer hypermethylation markers, **(b)** all cancer hypomethylation markers, **(c)** all tissue hypermethylation markers, and **(d)** all tissue hypomethylation markers. Note that hypermethylated (hypermethylated) read count refers to the count scaled by the sample sequencing depth, i.e., $\text{normalized hypermethylated read count in a marker for a sample} = 10^9 \frac{\text{Number of hypermethylated (hypomethylated) reads in a marker}}{\text{Number of reads in the sample}}$, whose unit is count per billion. Source data are provided as a Source Data file.

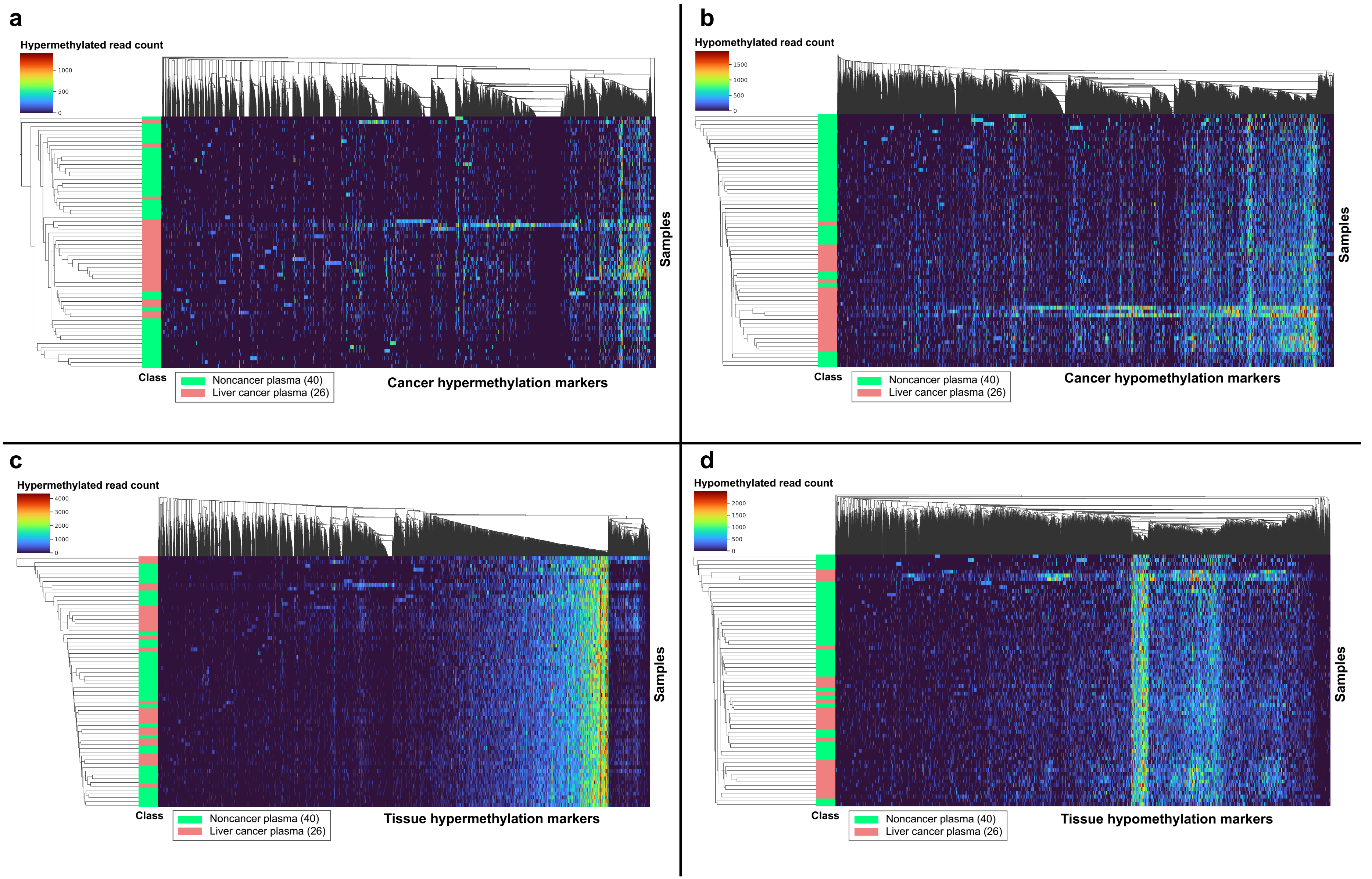

Supplementary Figure S4. Heatmap of four types of markers shown using a published WGBS dataset of 66 plasma samples (including 40 noncancer and 26 liver cancer samples)[27]. **(a)** all cancer hypermethylation markers, **(b)** all cancer hypomethylation markers, **(c)** all tissue hypermethylation markers, and **(d)** all tissue hypomethylation markers. Note that hypermethylated (hypermethylated) read count refers to the count scaled by the sample sequencing depth, i.e., $\text{normalized hypermethylated read count in a marker for a sample} = 10^9 \frac{\text{Number of hypermethylated (hypomethylated) reads in a marker}}{\text{Number of reads in the sample}}$, whose unit is count per billion. Source data are provided as a Source Data file.

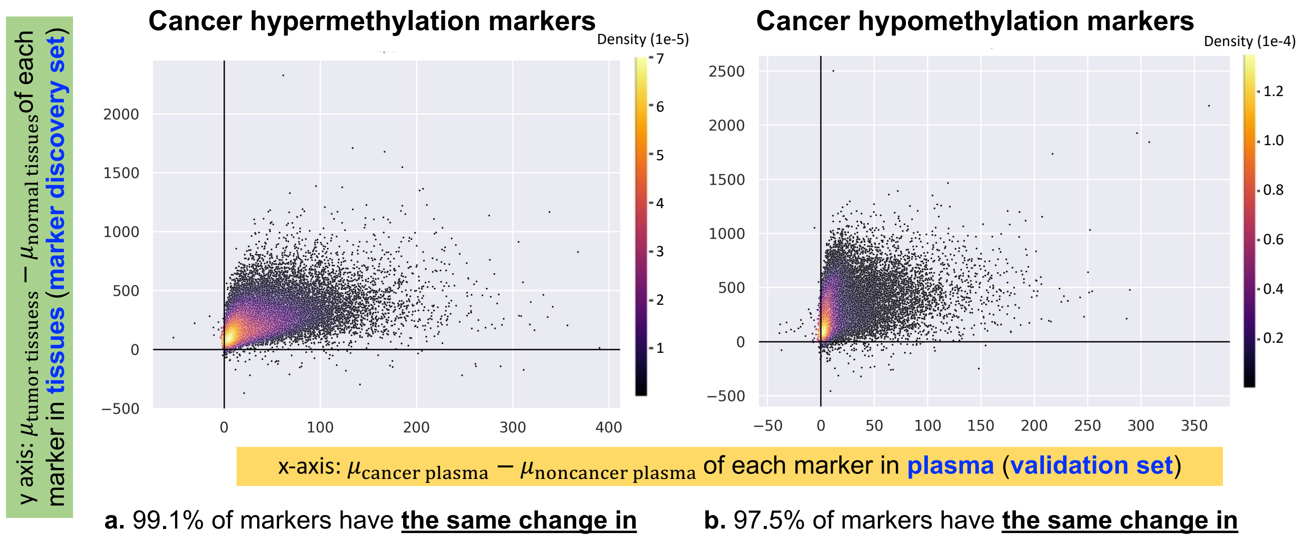

a. 99.1% of markers have **the same change in direction from case to control samples** for marker discovery set and validation set

b. 97.5% of markers have **the same change in direction from case to control samples** for marker discovery set and validation set

Note:

1. Each point in the scatter plots represents a marker.
2. Yellow color indicates higher density of points, black color lower density. For details see color bars.
3. $\mu_{\text{case samples}} - \mu_{\text{control samples}}$ is the change in the average tumor read count from case samples to control samples, where μ_{samples} is an average of the tumor read count among a group of samples.
4. **Marker discovery set:** RRBS data of 262 tissues, where cases are 131 tumors, controls are 131 normal tissues.
5. **Validation set:** cfMethyl-seq data of our plasma samples, where cases are 217 cancer plasma, controls are 161 noncancer plasma (i.e., 30 noncancer plasma samples used for marker discovery are removed from 191 all noncancer plasma samples).

Supplementary Figure S5. Density scatter plots of (a) all cancer hypermethylation and (b) all cancer hypomethylation markers, comparing the change (x coordinate) of hypermethylated (hypomethylated) read counts from tumors to normal tissues, in the marker discovery set, and the change (y coordinate) of hypermethylated (hypomethylated) read counts from cancer plasma to noncancer plasma in the validation set. Each marker is a point in the scatter plot. If a marker is located in the First Quadrant (i.e., x>0 and y>0), it means this marker has the same change in direction from case to control samples for the marker discovery set and the validation set, indicating that the tumor signal identified in tissues used for marker discovery set exists in plasma of the validation set. As you can see from two scatter plots, the majority of cancer hypermethylation/hypomethylation markers have tumor signals. For details see the explanation in the note of the figure. Source data are provided as a Source Data file.

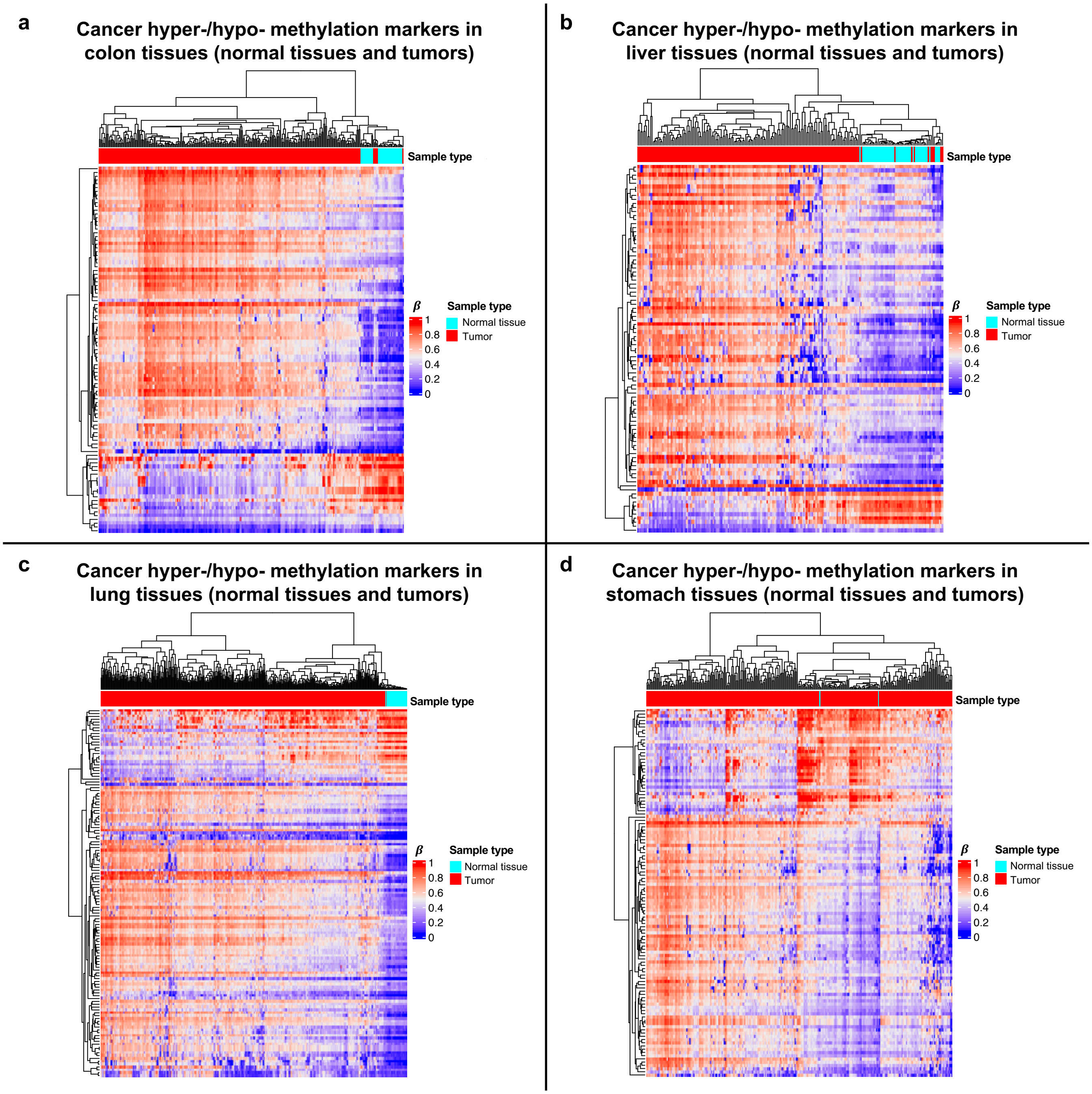

Supplementary Figure S6. Heatmap of DNA methylation markers (i.e., the average β-value of 450K probes within the marker) shown using TCGA 450K data, for (a) colon tissues, (b) liver tissues, (c) lung tissues and (d) stomach tissues. We used the union set of the top 200 cancer-specific hypermethylation and the top 200 cancer-specific hypomethylation markers of each cancer type for the heatmaps. The heatmaps show only those markers that overlap with 450K probe regions. Source data are provided as a Source Data file.

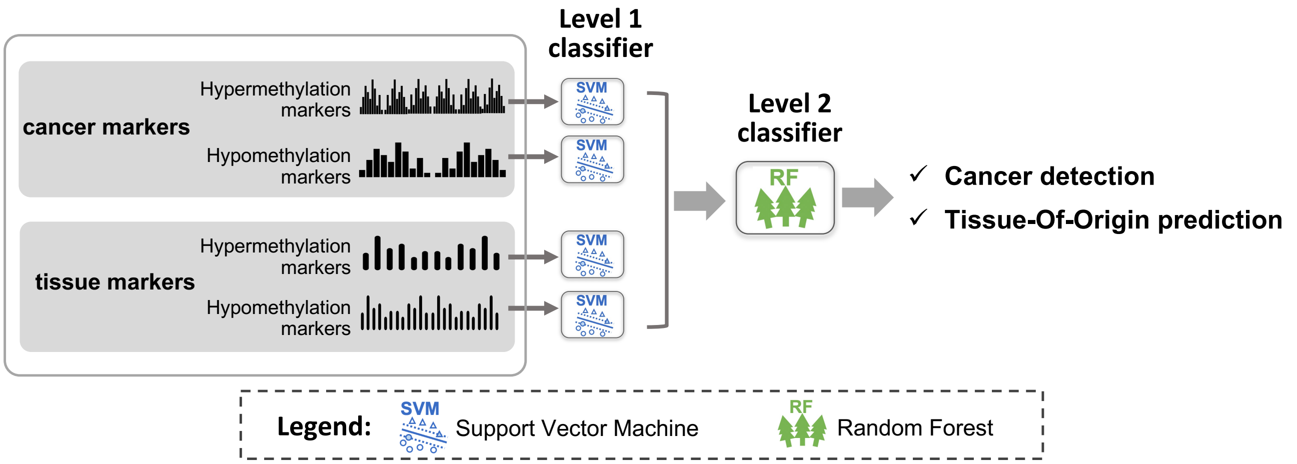

Supplementary Figure S7. Flowchart of the integrative learning framework using multi-feature data for cancer detection (using binary classifiers) and tissue-of-origin prediction (using multi-class classifiers).

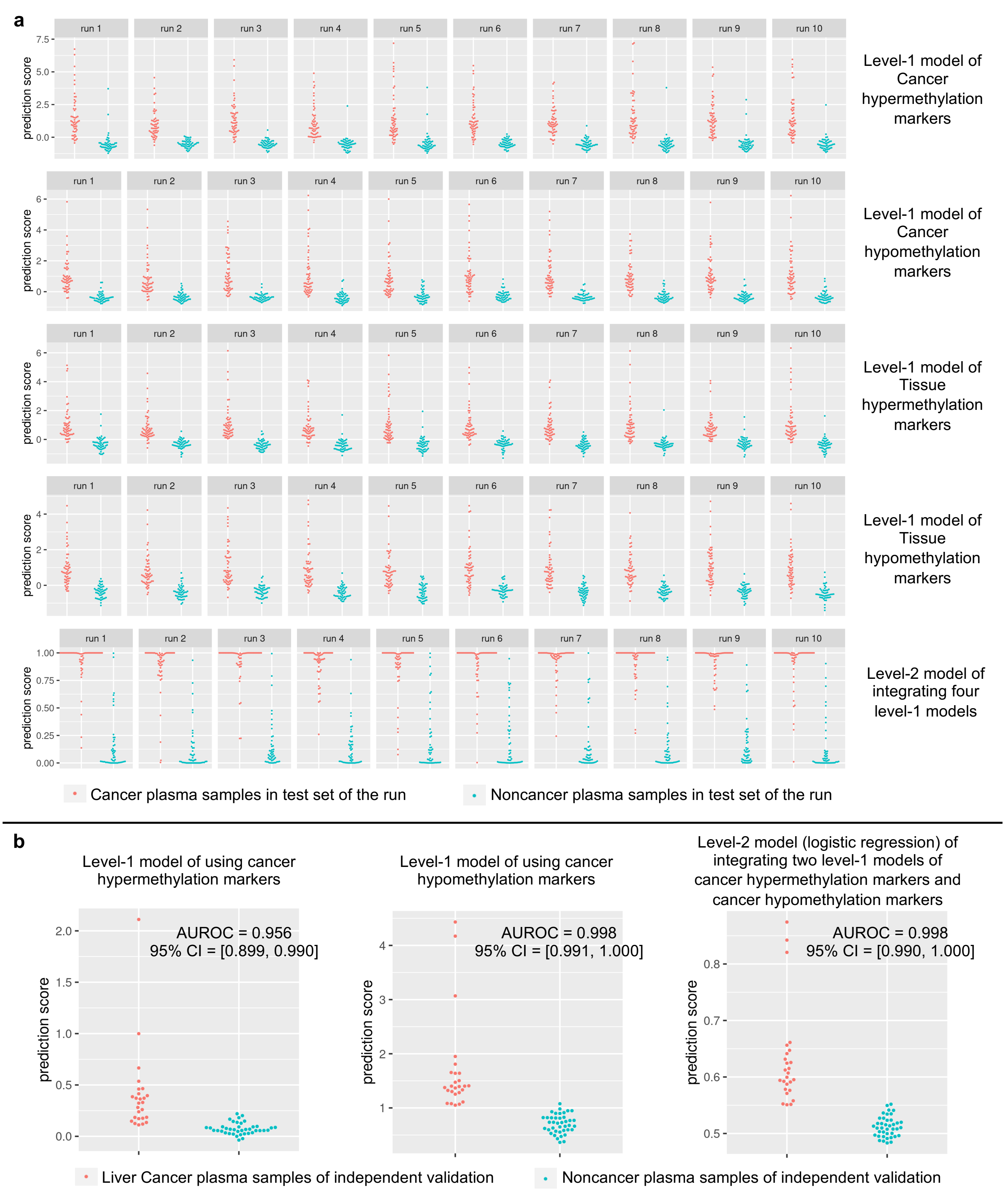

Supplementary Figure S8. Prediction scores of (a) the level-1 models of four methylation marker types and the level-2 model that integrates four marker types, shown on cfMethyl-Seq data of the test plasma samples in the 10-random-split validation; and **(b)** the independent dataset from level-1 cancer hypermethylation and cancer hypomethylation models, and level-2 linear model (logistic regression) that integrates two level-1 models. The prediction score for each plasma sample in the independent dataset was obtained by averaging the 10 prediction scores from the 10 cancer detection classifiers, which were trained from the 10 random splits of our cfMethyl-Seq data. The CI of the AUROC was calculated using bootstrap (n = 1000). Source data are provided as a Source Data file.

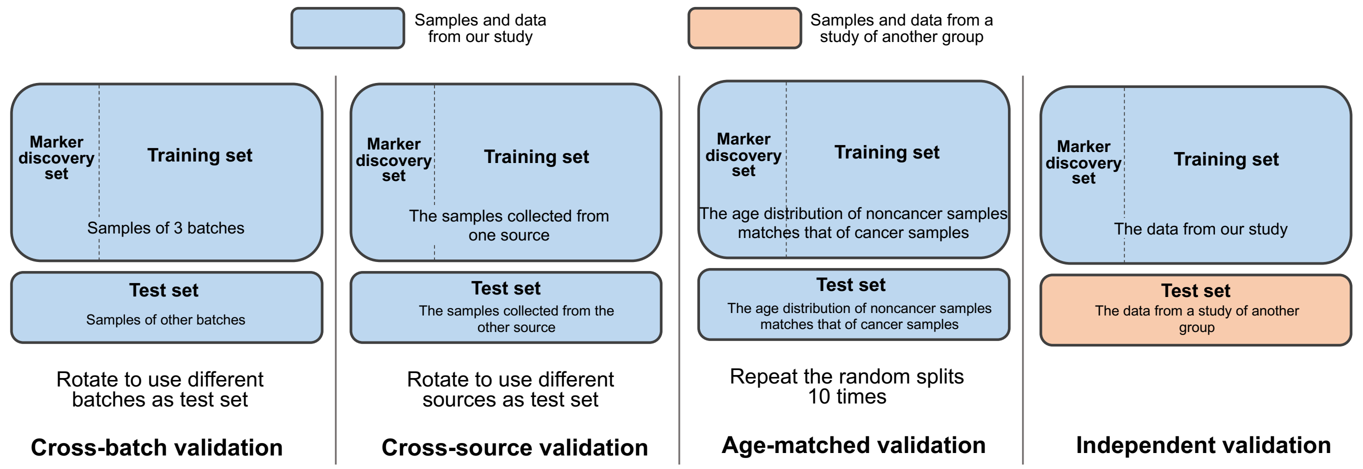

Supplementary Figure S9. Plasma sample splitting schema for the four validation strategies to evaluate the robustness of the model against potential confounders. These four validation strategies include cross-batch validation, cross-source validation, age-matched validation, and independent validation. In each validation, samples are split into three sets: marker discovery set, training set, and test set.

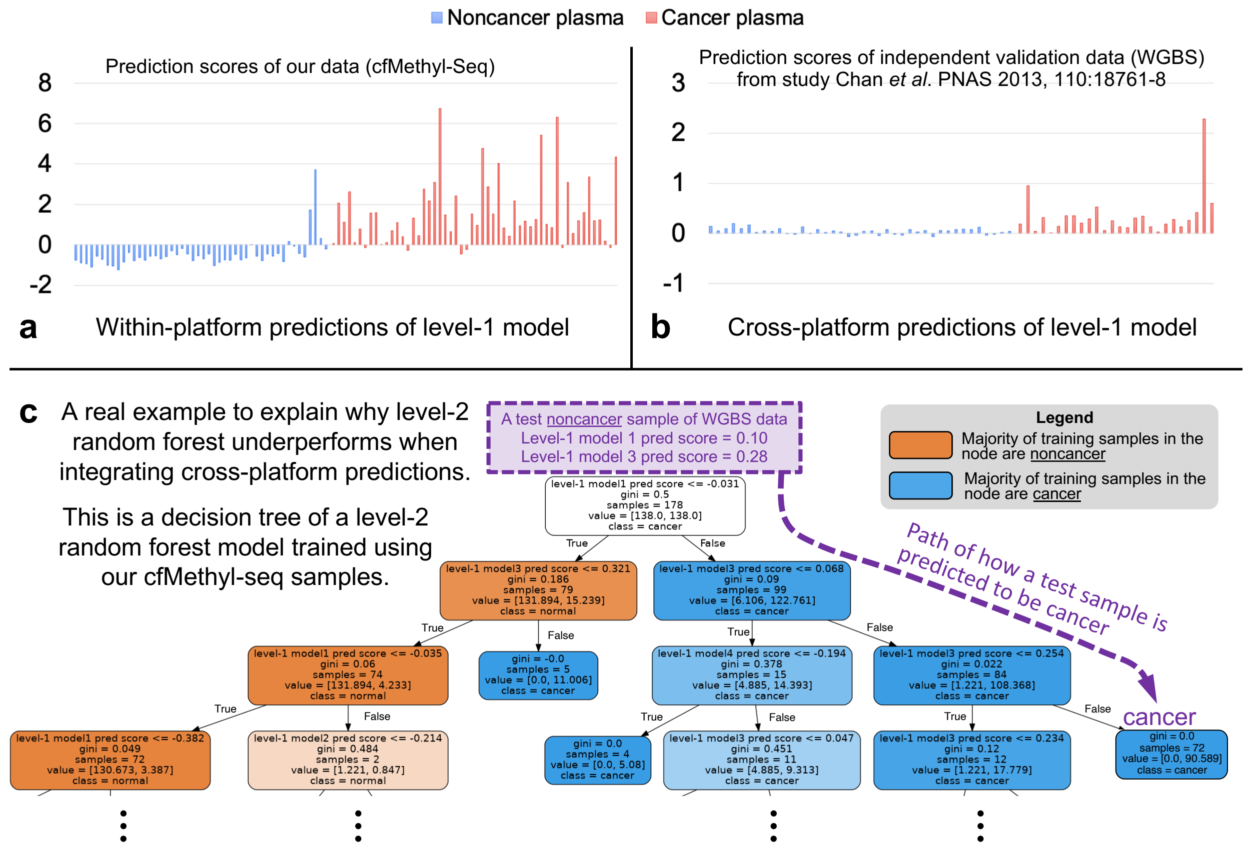

d Performance of different level-2 models that integrate different marker types and three popular classifiers, for cross-platform predictions and within-platform predictions.

Level-2 classifier	AUROC (independent data, cross-platform predictions)	AUROC (our data, within-platform predictions)	
	Integrating two level-1 models (i.e., two cancer marker types*)	Integrating two level-1 models (i.e., two cancer marker types*)	Integrating four level-1 models (i.e., all four marker types)
**Level-2 Logistic Regression**	0.998	0.964	0.971
**Level-2 Linear SVM**	0.998	0.964	0.971
**Level-2 Random Forest**	0.967	0.957	0.974

Supplementary Figure S10. The magnitudes of the output prediction scores of the level-1 model are different when using data from different platforms, which results in the underperformance of level-2 random forest for cross-platform predictions. Here we use cancer hypermethylation markers as an example of a level-1 model. Using the data generated by the cfMethyl-seq platform as the training set, **(a)** the prediction scores on the testing data of the same platform (i.e., cfMethyl-seq), and **(b)** the prediction scores of the testing data of a different platform (i.e., WGBS) in a different study, containing 66 plasma samples (40 noncancer and 26 liver cancer samples)[27]. As shown above, the magnitude of the prediction scores in (a) and (b) are different. However, the prediction scores in (a) and (b) can differentiate noncancer and cancer plasma samples well, yielding the high AUROCs for the level-1 model. Source data of (a) and (b) are provided as a Source Data file. **(c) A real example to explain why random forest underperforms as the level-2 classifier for the cross-platform predictions.** A tree of the level-2 random forest classifier is shown, which is trained by using our cfMethyl-Seq data. Because the magnitude of prediction scores produced by the level-1 models for two platforms (WGBS and cfMethyl-seq) are different, as shown in (a-b), the threshold in each tree in the random forest that fits the magnitude of level-1 models' prediction scores of cfMethyl-seq samples could lead to a wrong classification when using the level-1 models' prediction scores of a test sample from the WGBS platform. See the example in the purple box and purple dotted line in (c). **(d) The performance of different level-2 classifiers, trained by cfMethyl-seq data (75% samples), on the independent WGBS data and the left-out (25% samples) *cfMethyl-seq* data.** For validation on the independent WGBS data, linear models outperformed the random forest model, because the random forest model suffers from the cross-platform data scale inconsistency. For the validation on the cfMethyl-seq data, if only two types of features were used, the level-2 linear models can achieve similar performance as the best level-1 models, and when we integrate all four types of features, the level-2 linear models both achieve higher performance than all level-1 models, highlighting the ability of ensemble learning to extract complementary information from distinct types of features. The best level-2 results were achieved by the level-2 random forest model (AUROC=0.974), which was reported in the main text.

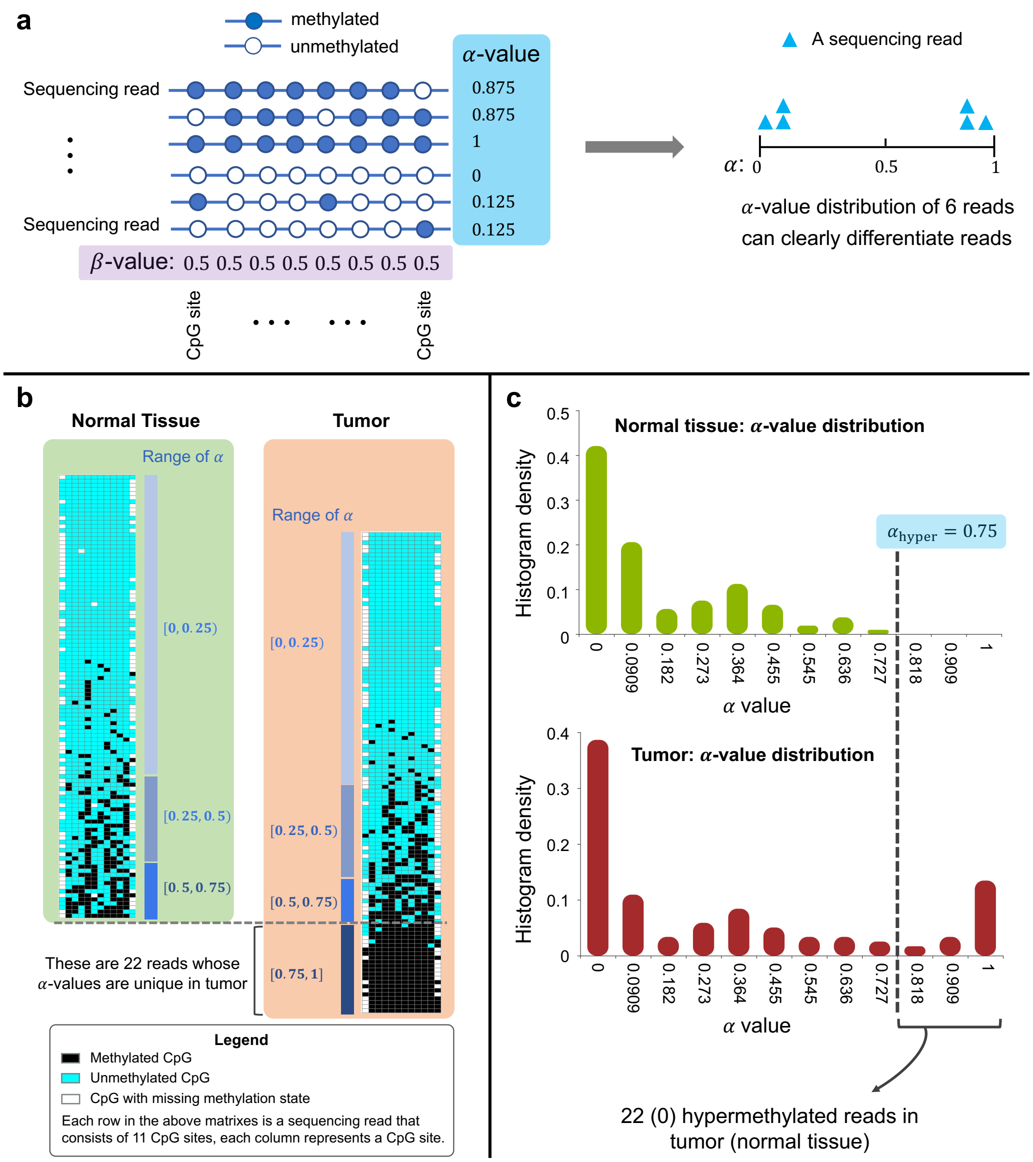

Supplementary Figure S11. Read-based marker discovery. (a) Conceptual illustration of using α-value to separate reads based on their pervasive methylation statuses. Each line represents a sequencing read and each dot represents a CpG site, where solid blue dots represent methylated CpG sites, and hollow blue dots represent unmethylated CpG sites. The α-value of a read (methylation value averaged across all CpG sites in this read, horizontal direction) can exploit the pervasive nature of methylation to sensitively separate methylated from unmethylated reads, while the commonly used β-value of a CpG site (methylation level of a CpG site averaged across all reads, vertical direction) cannot. As illustrated in the example in (a), all 8 CpG sites have the same β-value 0.5; while the α-value of each sequencing read can clearly differentiate the 3 pervasively methylated reads (i.e., their α-values are close to 1) from the 3 pervasively unmethylated reads (their α-values are close to 0). **(b) A real example to illustrate the read-based marker discovery method, using a lung cancer hypermethylation marker (chr10:22625738-22625835) with 12 CpG sites.** The methylation states of all sequencing reads falling onto the marker region are shown in a tumor and its adjacent normal tissue. Each read has an α-value reflecting its methylation pattern. For example, both tumor and normal tissues have reads with α-values in $[0, 0.25)$, but 22 reads with α-values in $[0.75, 1]$ are unique to the tumor only. These reads ($\alpha \geq 0.75$) can be defined as tumor reads which are highly methylated in tumor and cannot be found in normal tissue. Note that because of the enzymatic digestion, only 11 out of the 12 CpG sites contain methylation information (with respect to the forward strand, the 3' CpG site of a forward mapped read or the 5' CpG site of a reverse mapped read has no methylation information). **(c) Identifying an α_{hyper} threshold from the α-value distributions.** Using the α-value distributions of the tumor and normal tissues from (b), we can identify a threshold α_{hyper} (e.g. 0.75) to define "hypermethylated reads" that occur mostly in tumor but not normal samples.

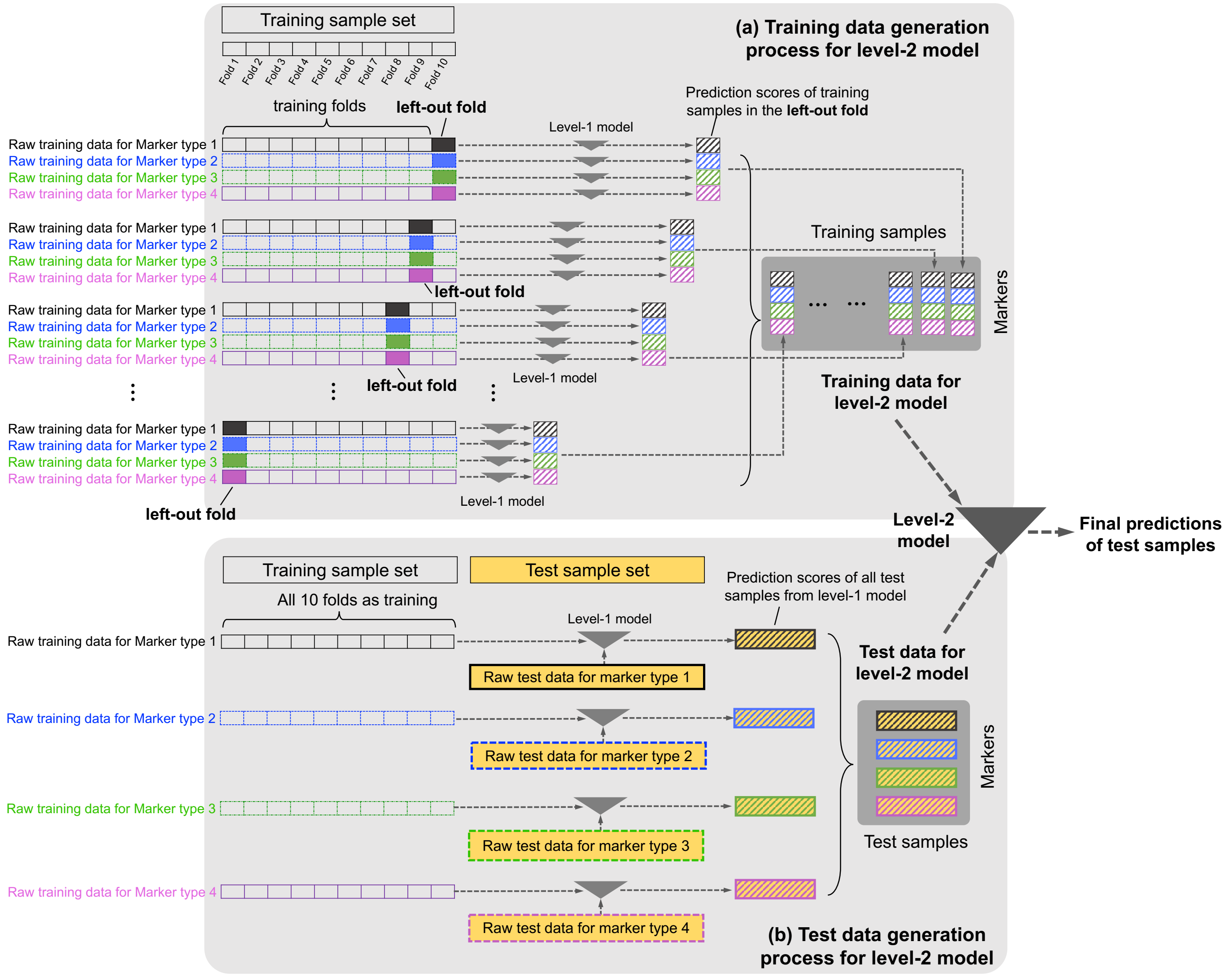

Supplementary Figure S12. Illustrative flowchart of the learning and prediction process of the two-level stacked ensemble model: (a) Generate training data for level-2 model. We split the training samples into 10 equal-size folds. We then use 9 folds as training folds to train the level-1 model and make the predictions for the left-out 1 fold. We repeat this process for each left-out fold, until every training sample can receive a prediction score from a level-1 model. Then the prediction scores of the training samples are used as the training data for level-2 model. **(b)** Generate test data for level-2 model. We use the data of all training samples to train one level-1 model and make predictions for the test samples. Then the prediction scores of the test samples are used as the test data for level-2 model.

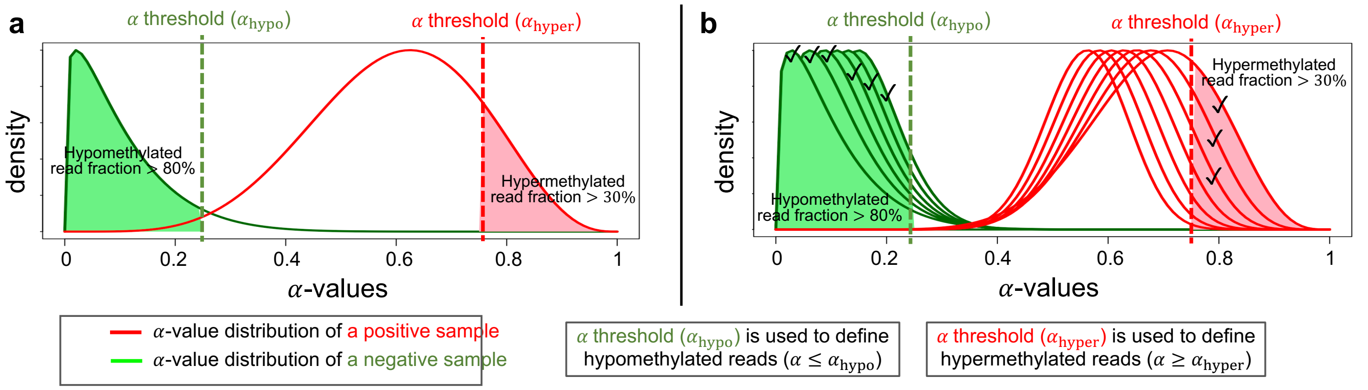

Supplementary Figure S13. Conceptual illustration of the read-based methylation marker discovery method. (a). The principle illustrated with the α-value distributions of a positive sample and a negative sample. The method aims to find two α-value thresholds (α_{hyper} and α_{hypo}) that define hyper- and hypo-methylated reads that satisfy three criteria: (1) the two α-value thresholds are far from each other (e.g., $\alpha_{\mathrm{hyper}} - \alpha_{\mathrm{hypo}} = 0.5$ in **(a)**); (2) the majority (e.g., >80% in **(a)**) of reads in the negative sample are hypo-methylated; (3) a significant fraction (e.g., >30% in **(a)**) of reads in the positive sample are hypermethylated. **(b)** Generalization of the principle to multiple positive samples and negative samples, where 6 negative samples and 7 positive samples are illustrated. We aim to find two α-value thresholds (α_{hyper} and α_{hypo}), satisfying three criteria: (1) the two α thresholds are far from each other (e.g., $\alpha_{\mathrm{hyper}} - \alpha_{\mathrm{hypo}} = 0.5$ in **(b)**); (2) in most negative samples (e.g., all 6 negative samples in **(b)**), the majority (e.g., >80% in **(b)**) of reads are hypo-methylated; (3) in a subset (e.g., 3) of positive samples, a significant fraction (e.g., >30% in **(b)**) of reads are hypermethylated. Each negative sample whose α-value distribution satisfies Criterion (2) and each positive sample whose α-value distribution satisfies Criterion (3) receives a tick symbol in **(b)**. In **(b)**, all 6 negative samples receive ticks and 3 out of 7 positive samples receive ticks.

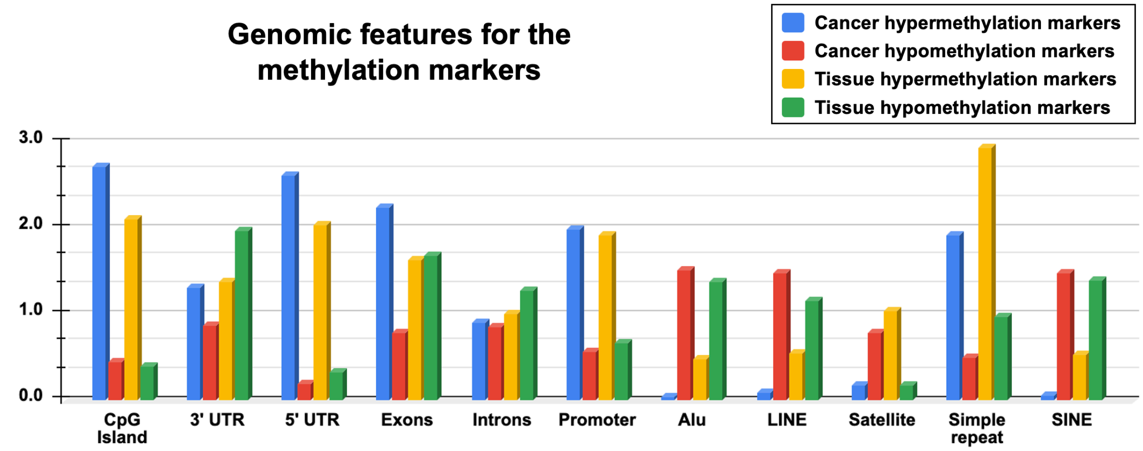

Supplementary Figure S14. Enrichment of genomic features. The bar charts show the enrichment fold changes of the following genomic features in the methylation markers: CpG Islands, 3' UTR, 5' UTR, exons, introns, promoters, and Repeat elements (Alu, LINE, Satellite, simple repeat, and SINE). The methylation marker types are cancer-specific hypermethylation and hypomethylation markers for cancer detection and tissue-specific hypermethylation and hypomethylation markers. Source data are provided as a Source Data file.

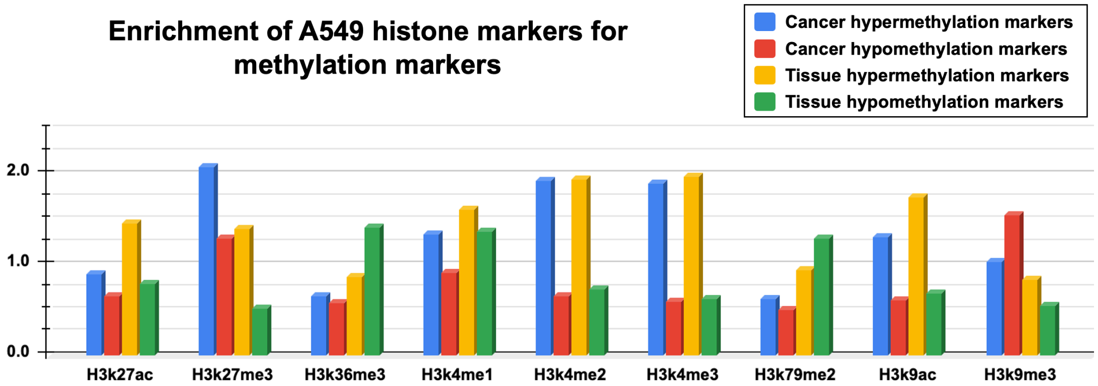

Supplementary Figure S15. Enrichment of A549 histone markers. The bar charts show the enrichment fold changes of A549 histone markers in methylation markers: H3k27ac, H3k27me3, H3k36me3, H3k4me1, H3k4me2, H3k4me3, H3k79me2, H3k9ac, and H3k9me3. The methylation marker types are cancer-specific hypermethylation and hypomethylation markers for cancer detection and tissue-specific hypermethylation and hypomethylation markers. Source data are provided as a Source Data file.

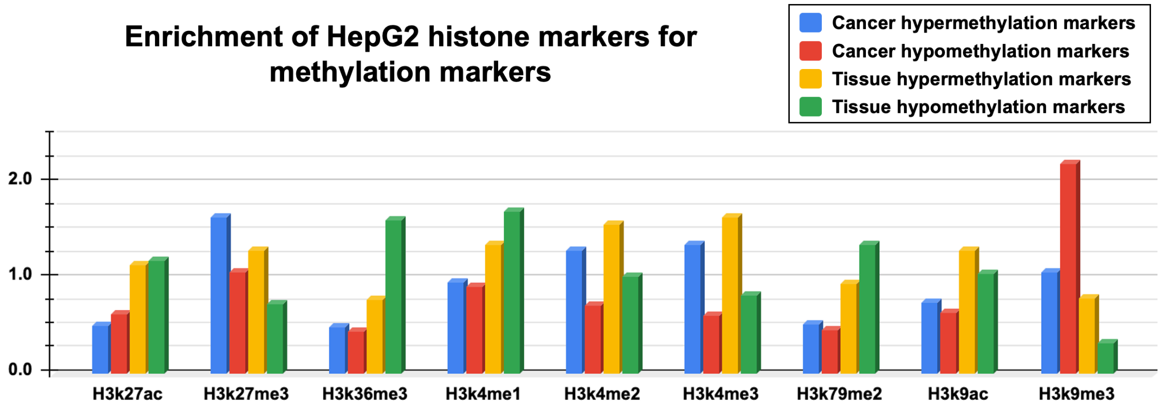

Supplementary Figure S16. Enrichment of HepG2 histone markers. The bar charts show the enrichment fold changes of HepG2 histone markers in the liver cancer methylation markers for: H3k27ac, H3k27me3, H3k36me3, H3k4me1, H3k4me2, H3k4me3, H3k79me2, H3k9ac, and H3k9me3. The methylation marker types are cancer-specific hypermethylation and hypomethylation markers for cancer detection and tissue-specific hypermethylation and hypomethylation markers. Source data are provided as a Source Data file.

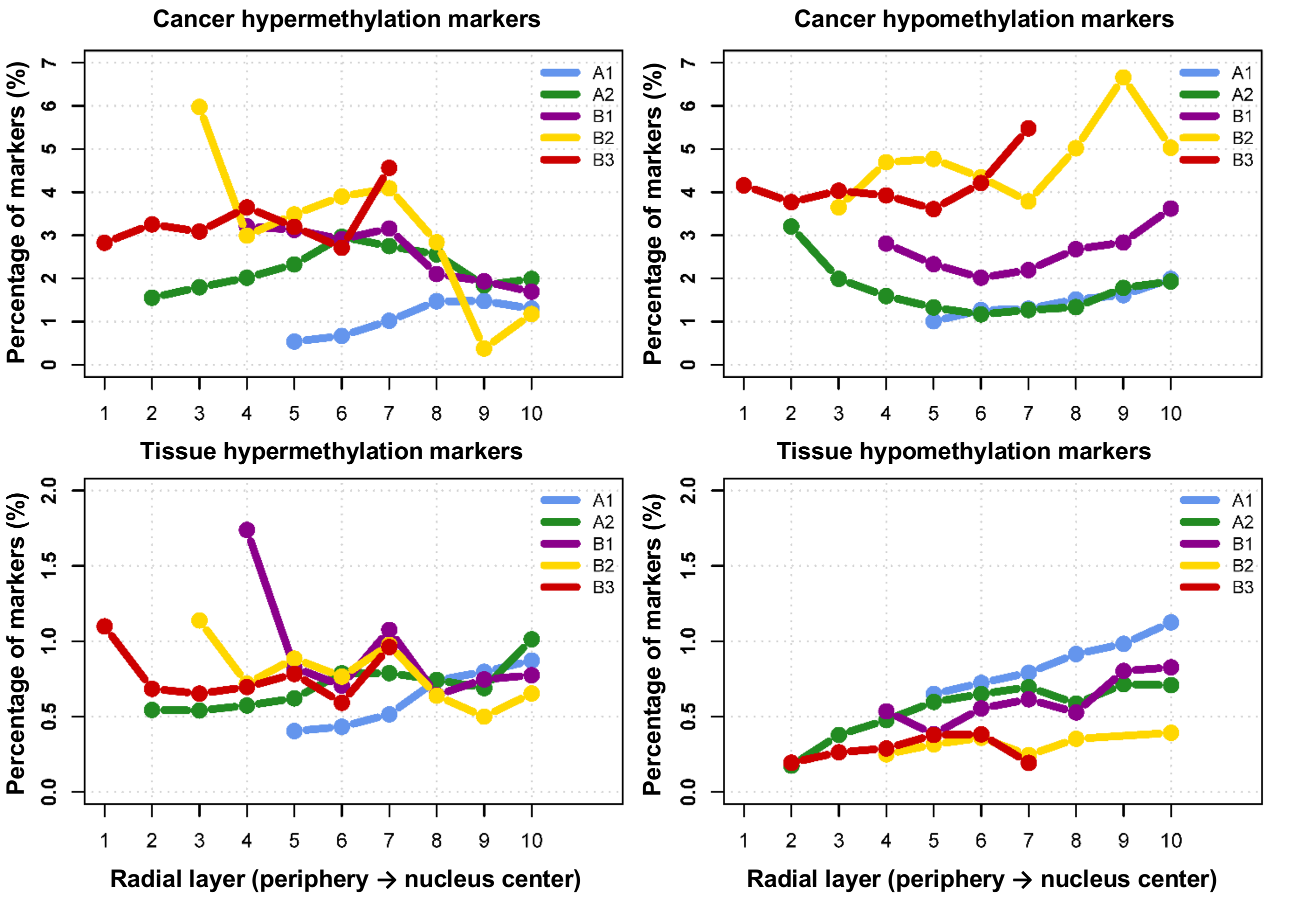

Supplementary Figure S17. Chromatin radial distribution of the tumor and tissue hyper/hypo methylated markers (100-kb resolution). The x-axis indicates ten concentric layers defined based on the GPSeq score (layer 10=nucleus, layer 1=periphery), and the y-axis indicates the percentage of markers, calculated as the number of markers divided by the number of all marker candidates in a 3D grid, where the 3D grids are defined by the intersection between ten radial layers and five subcompartments (A1, A2, B1, B2, and B3). The methylation marker types are cancer-specific hypermethylation and hypomethylation markers for cancer detection and tissue-specific hypermethylation and hypomethylation markers. Source data are provided as a Source Data file.

Supplementary References

1. Li, W. *et al.* CancerDetector: ultrasensitive and non-invasive cancer detection at the resolution of individual reads using cell-free DNA methylation sequencing data. *Nucleic Acids Res* **46**, e89 (2018).
2. Zeybel, M. *et al.* Multigenerational epigenetic adaptation of the hepatic wound-healing response. *Nat Med* **18**, 1369–77 (2012).
3. Angulo, P. *et al.* Liver Fibrosis, but No Other Histologic Features, Is Associated With Long-term Outcomes of Patients With Nonalcoholic Fatty Liver Disease. *Gastroenterology* **149**, 389–97.e10 (2015).
4. Hardy, T. *et al.* Plasma DNA methylation: a potential biomarker for stratification of liver fibrosis in non-alcoholic fatty liver disease. *Gut* **66**, 1321–1328 (2017).
5. Harrow, J. *et al.* GENCODE: The reference human genome annotation for the ENCODE project. *Genome Research* **22**, 1760–1774 (2012).
6. Casper, J. *et al.* The UCSC Genome Browser database: 2018 update. *Nucleic Acids Research* **46**, D762–D769 (2018).
7. Liberzon, A. *et al.* The Molecular Signatures Database Hallmark Gene Set Collection. *Cell Systems* **1**, 417–425 (2015).
8. Liberzon, A. *et al.* Molecular signatures database (MSigDB) 3.0. *Bioinformatics* **27**, 1739–1740 (2011).
9. Laugesen, A., Højfeldt, J. W. & Helin, K. Role of the polycomb repressive complex 2 (PRC2) in transcriptional regulation and cancer. *Cold Spring Harbor Perspectives in Medicine* **6**, (2016).
10. Mitchell, J. A. *et al.* Gene indexing: characterization and analysis of NLM's GeneRIFs. *AMIA Annu Symp Proc* 460–464 (2003).
11. Li, W. H. *et al.* Detection of OSR2, VAV3, and PPFIA3 Methylation in the Serum of Patients with Gastric Cancer. *Dis Markers* **2016**, (2016).
12. Tang, H. *et al.* Interaction of hsa-miR-381 and glioma suppressor LRRC4 is involved in glioma growth. *Brain Res* **1390**, 21–32 (2011).
13. Kumar, A., Gosipatala, S. B., Pandey, A. & Singh, P. Prognostic Relevance of SFRP1 Gene Promoter Methylation in Colorectal Carcinoma. *Asian Pac J Cancer Prev* **20**, 1571–1577 (2019).
14. Roperch, J. P. *et al.* Aberrant methylation of NPY, PENK, and WIF1 as a promising marker for blood-based diagnosis of colorectal cancer. *BMC Cancer* **13**, (2013).
15. Ahn, J. *et al.* FOXC2 and CLIP4 : a potential biomarker for synchronous metastasis of ≤7-cm clear cell renal cell carcinomas. *Oncotarget* **7**, 51423–51434 (2016).
16. Huang, C., He, C., Ruan, P. & Zhou, R. TSPYL5 activates endoplasmic reticulum stress to inhibit cell proliferation, migration and invasion in colorectal cancer. *Oncol Rep* **44**, 449–456 (2020).
17. Snellenberg, S. *et al.* Methylation-mediated repression of PRDM14 contributes to apoptosis evasion in HPV-positive cancers. *Carcinogenesis* **35**, 2611–2618 (2014).
18. Yamada, Y., Kurata, A., Fujita, K. & Kuroda, M. Fascin as a useful marker for cancer-associated fibroblasts in invasive lung adenocarcinoma. *Medicine* **100**, e27162 (2021).
19. Tran, M. H. *et al.* NEDD4-induced degradative ubiquitination of phosphatidylinositol 4-phosphate 5-kinase α and its implication in breast cancer cell proliferation. *J Cell Mol Med* **22**, 4117–4129 (2018).
20. Li, X. R. *et al.* CK5/6, EGFR, Ki-67, cyclin D1, and nm23-H1 protein expressions as predictors of pathological complete response to neoadjuvant chemotherapy in triple-negative breast cancer patients. *Med Oncol* **28 Suppl 1**, (2011).
21. Aktary, Z. *et al.* Plakoglobin interacts with and increases the protein levels of metastasis suppressor Nm23-H2 and regulates the expression of Nm23-H1. *Oncogene* **29**, 2118–2129 (2010).

22. Girelli, G. *et al.* GPSeq reveals the radial organization of chromatin in the cell nucleus. *Nature Biotechnology* **38**, 1184–1193 (2020).

23. Rao, S. S. P. *et al.* A 3D Map of the Human Genome at Kilobase Resolution Reveals Principles of Chromatin Looping. *Cell* (2014) doi:10.1016/j.cell.2014.11.021.

24. Xiong, K. & Ma, J. Revealing Hi-C subcompartments by imputing inter-chromosomal chromatin interactions. *Nature Communications* **10**, (2019).

25. Aggarwal, C. C. *Data Classification: Algorithms and Applications*. (Chapman & Hall/CRC, 2014).

26. Wolpert, D. H. Stacked generalization. *Neural networks* **5**, 241–259 (1992).

27. Chan, K. C. A. *et al.* Noninvasive detection of cancer-associated genome-wide hypomethylation and copy number aberrations by plasma DNA bisulfite sequencing. *Proc Natl Acad Sci U S A* **110**, 18761–8 (2013).
