# Supplementary material for: Cost-effective methylome sequencing of cell-free DNA for accurately detecting and locating cancer
Source: Nat Commun. 2022 Sep 29;13:5566. doi: 10.1038/s41467-022-32995-6 (PMC9522828; doi:10.1038/s41467-022-32995-6)
Supplement: Supplementary file 2 — Description of Additional Supplementary Files [file 41467_2022_32995_MOESM2_ESM.pdf]

File Name: Supplementary Data 1
Description: cancer-specific hypermethylation markers used for cancer detection, hg19 coordinates

File Name: Supplementary Data 2
Description: cancer-specific hypomethylation markers used for cancer detection, hg19 coordinates

File Name: Supplementary Data 3
Description: cancer-specific hypermethylation markers used for TOO prediction, hg19 coordinates

File Name: Supplementary Data 4
Description: cancer-specific hypomethylation markers used for TOO prediction, hg19 coordinates

File Name: Supplementary Data 5
Description: tissue-specific hypermethylation markers, hg19 coordinates

File Name: Supplementary Data 6
Description: tissue-specific hypomethylation markers, hg19 coordinates

File Name: Supplementary Data 7
Description: GeneRIF for the top 50 cancer hypermethylation markers for each cancer type

File Name: Supplementary Data 8
Description: GeneRIF for the top 50 cancer hypomethylation markers for each cancer type

File Name: Supplementary Data 9
Clinical info for cfDNA samples

File Name: Supplementary Data 10
Description: Clinical info for solid tissue samples

File Name: Supplementary Data 11
Oligos used in this study

File Name: Supplementary Data 12
Description: Mapping statistics for cfDNA and solid tissue samples

File Name: Supplementary Data 13
Description: Column explanations for mapping statistics
