# Supplementary material for: Cost-effective methylome sequencing of cell-free DNA for accurately detecting and locating cancer
Source: Nat Commun. 2022 Sep 29;13:5566. doi: 10.1038/s41467-022-32995-6 (PMC9522828; doi:10.1038/s41467-022-32995-6)
Supplement: Supplementary file 16 — Reporting Summary [file 41467_2022_32995_MOESM16_ESM.pdf]

nature research

Corresponding author(s): Xianghong Jasmine Zhou, Wenyuan Li, Xiaohui Ni, Steven-Huy Han, Vatche Agopian

Last updated by author(s): August 11, 2022

Reporting Summary

Nature Research wishes to improve the reproducibility of the work that we publish. This form provides structure for consistency and transparency in reporting. For further information on Nature Research policies, see our Editorial Policies and the Editorial Policy Checklist.

Statistics

For all statistical analyses, confirm that the following items are present in the figure legend, table legend, main text, or Methods section.

n/a	Confirmed	
☐	☒	The exact sample size (*n*) for each experimental group/condition, given as a discrete number and unit of measurement
☐	☒	A statement on whether measurements were taken from distinct samples or whether the same sample was measured repeatedly
☐	☒	The statistical test(s) used AND whether they are one- or two-sided<br>*Only common tests should be described solely by name; describe more complex techniques in the Methods section.*
☒	☐	A description of all covariates tested
☒	☐	A description of any assumptions or corrections, such as tests of normality and adjustment for multiple comparisons
☐	☒	A full description of the statistical parameters including central tendency (e.g. means) or other basic estimates (e.g. regression coefficient) AND variation (e.g. standard deviation) or associated estimates of uncertainty (e.g. confidence intervals)
☒	☐	For null hypothesis testing, the test statistic (e.g. *F*, *t*, *r*) with confidence intervals, effect sizes, degrees of freedom and *P* value noted<br>*Give P values as exact values whenever suitable.*
☒	☐	For Bayesian analysis, information on the choice of priors and Markov chain Monte Carlo settings
☒	☐	For hierarchical and complex designs, identification of the appropriate level for tests and full reporting of outcomes
☒	☐	Estimates of effect sizes (e.g. Cohen's *d*, Pearson's *r*), indicating how they were calculated

Our web collection on statistics for biologists contains articles on many of the points above.

Software and code

Policy information about availability of computer code

Data collection	No software was used for data collection.
Data analysis	All software and packages used in our analyses are open-sourced.<br><br>Bismark v0.18.2<br>python3 3.8.5 (sklearn 0.23.2)<br>trim_galore 0.4.4<br>Umi-Grinder v0.0.1<br><br>Software code is available at https://github.com/jasminezhoulab/cfMethyl-Seq and can be freely used for educational and research purposes by non-profit institutions and U.S. government agencies. For information on the use for a commercial purpose or by a commercial or for-profit entity, please contact Professor Xianghong Jasmine Zhou.

For manuscripts utilizing custom algorithms or software that are central to the research but not yet described in published literature, software must be made available to editors and reviewers. We strongly encourage code deposition in a community repository (e.g. GitHub). See the Nature Research guidelines for submitting code & software for further information.

Data

Policy information about availability of data

All manuscripts must include a data availability statement. This statement should provide the following information, where applicable:

- Accession codes, unique identifiers, or web links for publicly available datasets
- A list of figures that have associated raw data
- A description of any restrictions on data availability

Raw sequencing data generated in this study (RRBS data from 328 solid tissue samples and cfMethyl-seq data from 479 plasma samples) have been deposited into the European Genome-Phenome Archive under accession code EGAS00001006020 (https://ega-archive.org/studies/EGAS00001006020). The data are available under controlled access due to the sensitive nature of genome sequencing data, and access can be obtained by contacting the corresponding authors. The corresponding authors will generally respond to requests within three days. The remaining data are available within the Article, Supplementary Information, or Source data. Data from the study EGAS00001000566 (https://ega-archive.org/studies/EGAS00001000566, 40 noncancer and 26 liver cancer WGBS samples) were used for independent validation and are associated with Supplementary Fig. S4, S8b and S10. TCGA 450k array data were obtained from https://cancergenome.nih.gov/ and are associated with Supplementary Fig. S6. Reference genome hg19 (GRCh37 (GCA 000001405.1) was used for mapping samples. Source data are provided with this paper.

Field-specific reporting

Please select the one below that is the best fit for your research. If you are not sure, read the appropriate sections before making your selection.

[x] Life sciences [ ] Behavioural & social sciences [ ] Ecological, evolutionary & environmental sciences

For a reference copy of the document with all sections, see nature.com/documents/nr-reporting-summary-flat.pdf

Life sciences study design

All studies must disclose on these points even when the disclosure is negative.

Sample size	We collected 479 cfDNA samples from healthy subjects and those with 5 cancer subtypes. Additionally we sourced 328 solid tissue samples from the 5 cancer types. No sample size collection was performed; we sourced as many samples as possible from companies and UCLA hospital resources, essentially exhausting their supply.
Data exclusions	We excluded cfDNA samples that were collected prior to 2017 or were the wrong cancer subtype (not LUAD, LUSC, COAD, STAD, or LIHC), or were known to have undergone cancer treatment. For solid tissue samples, we removed samples that did not show flat CNV (normal) or obvious CNV (cancer), before any marker selection occurred. Old plasma samples were not used due to degradation concerns and outdated handling procedures. Samples from individuals who had undergone cancer treatment were removed because the goal of our method is to be used as a screening tool, prior to treatment. Solid tissue samples were excluded due to CNV profiles to remove samples that had obvious contamination (normal samples that had obvious CNV are not truly normal) or low tumor burden (tumor samples that had flat CNV may have been biopsied from non-tumor parts of the tissue). Using either of these cases would interfere with some of our marker selection processes that require comparisons between matched normal and tumor tissues.<br>In our revision, we further filtered our plasma samples to remove samples that had low coverage (<15x) and/or had low bisulfite conversion rate (<98.7%). This reduced from 479 to 408 samples. Data for all 479 samples will still be made available.
Replication	We repeated our marker generation, training, and testing 10 times to ensure reproducibility. There was a low standard deviation of results across the 10 runs, and selected markers were similar.
Randomization	Training and testing sets were balanced to be similar ages, genders, and stage/subtype breakdown. The training and testing sets were split 10 times, each time the training set was 75% of the samples and the testing set was the remaining 25%. The training and testing sets were balanced for covariates (age, gender, stage, cancer type) as much as possible. For example, if there were 4 stage IV liver cancer samples all similar ages, 3 were randomly selected to be put into the training set and 1 for the testing set. Normal samples were placed into 10-year age brackets and 75% of each bracket was randomly assigned to the training set and the remainder to the testing set. In cases where this was not possible, e.g. if there was only one stage III stomach cancer sample, it was randomly placed in either the training or testing set in each of the ten runs.
Blinding	No blinding was performed. This study is the first step in developing a screening tool using our ensemble learning approach, and knowledge of the subtypes/health status was necessary to evaluate the methods. Healthy cfDNA samples were needed to determine the initial marker set. Knowledge of stage/age/subtype was needed to fairly split the samples into training/testing.

Reporting for specific materials, systems and methods

We require information from authors about some types of materials, experimental systems and methods used in many studies. Here, indicate whether each material, system or method listed is relevant to your study. If you are not sure if a list item applies to your research, read the appropriate section before selecting a response.

Materials & experimental systems

n/a	Involved in the study
☒	☐ Antibodies
☒	☐ Eukaryotic cell lines
☒	☐ Palaeontology and archaeology
☒	☐ Animals and other organisms
☐	☒ Human research participants
☒	☐ Clinical data
☒	☐ Dual use research of concern

Methods

n/a	Involved in the study
☒	☐ ChIP-seq
☒	☐ Flow cytometry
☒	☐ MRI-based neuroimaging

Human research participants

Policy information about studies involving human research participants

Population characteristics	The age, gender, and health status (cancer subtype or non-cancer) are known for the 479 plasma samples. If cancer, the stage is known. All cancer samples are untreated. Non-cancer samples ranged from 21 to 87 years old with a median age of 50. 33% of non-cancer samples were female. Cancer samples ranged from 29 to 86 years old with a median age of 65. 36% of cancer samples were female.
Recruitment	Plasma samples were collected from UCLA hospitals or bought from commercial sources. For commercial sources, samples were not purchased if they were treated, the incorrect subtype (ie not LUAD, LUSC, COAD, STAD, or LIHC), or were missing age, stage, treatment, or gender information. Normal samples from commercial sources were specifically collected for us, and had no cancer in their medical histories. For UCLA hospitals, samples were collected from TPCL, IPH, or UCLA hospitals from patients receiving care at UCLA hospitals. Samples were required to be non-cancer or from LUAD, LUSC, COAD, STAD, or LIHC cancer subtypes and without treatment. Non-cancer samples sourced from UCLA hospitals tend to be older and have diseases other than cancer (e.g. cirrhosis) because these individuals came to UCLA seeking treatment or routine care (e.g. a colonoscopy). Cancer samples collected from UCLA meet the criteria of the IRBs associated with this study (IRB#19-000618, IRB#19-000230, IRB#19-001488, IRB#16-000659, IRB#17-000985), and may differ from commercially sourced samples in ethnicity, stage, and age breakdown due to the populations associated with our clinical collaborators. For example, liver cancer samples sourced at UCLA who are patients of V.A. tend to be early stage, lung cancer samples sourced at UCLA who are patients of D.A. tend to be early stage, and lung cancer samples sourced at UCLA who are patients of E.G. tend to be late stage.
Ethics oversight	The IRB at UCLA approved this study. Informed consent was obtained from all subjects. IRBs associated with this study are IRB#19-000618, IRB#19-000230, IRB#19-001488, IRB#16-000659, IRB#17-000985. Participants were not compensated for participation in this study.

Note that full information on the approval of the study protocol must also be provided in the manuscript.
